# Souporcell3: robust demultiplexing for high-donor single-cell RNA-seq datasets

**DOI:** 10.1093/bioinformatics/btag117

**Published:** 2026-03-10

**Authors:** Minindu Weerakoon, Hai Vu, Reza Behboudi, Haynes Heaton

**Affiliations:** Department of Computer Science and Software Engineering, Auburn University, Auburn, AL 36849, United States; Department of Computer Science and Software Engineering, Auburn University, Auburn, AL 36849, United States; Department of Computer Science and Software Engineering, Auburn University, Auburn, AL 36849, United States; Department of Computer Science and Software Engineering, Auburn University, Auburn, AL 36849, United States

## Abstract

**Motivation:**

Accurate demultiplexing of pooled single-cell RNA-seq (scRNA-seq) data is critical for large-scale studies. However, existing methods like vireo, while effective up to ∼16 donors, often struggle with poor clustering due to local optima as donor numbers rise. In high-donor scenarios, overlapping genotypes, a dense genotype space, and increased doublet formation make demultiplexing challenging, requiring methods that are robust to sparse, high-dimensional data and maintain reliable accuracy even as sample complexity grows.

**Results:**

We present an enhanced version of souporcell capable of demultiplexing up to 64 donors. The method uses 10× merge for initialization, K-Harmonic Means for robust clustering, and iterative refinement with reinitialization of low-quality clusters and locking of high-quality ones. Compared to vireo, overclustered vireo, and the original souporcell, our approach completely eliminates incorrectly merged clusters and achieves consistently high Adjusted Rand Index (ARI) scores across various doublet rates, demonstrating improved accuracy and scalability.

**Availability and implementation:**

Souporcell3 is freely available under the MIT open-source license at https://github.com/wheaton5/souporcell.

## 1 Introduction

Multiplexing cells from multiple individuals into a single scRNA-seq sample has become a popular experimental design because it reduces costs, avoids batch effects, increases statistical power, improves doublet detection, and allows us to measure and remove ambient RNA (Heaton *et al.* 2020, Young and Behjati 2020). Demultiplexing these samples was originally enabled by demuxlet (Xu *et al.* 2019) which required a priori knowledge of the genotypes of every individual in the mixture. If this information was not available, additional costly DNA sequencing of each sample was required. Later, souporcell (Heaton *et al.* 2020) and vireo (Huang *et al.* 2019) were developed to demultiplex cells by genotype without prior knowledge of their genotypes using various sparse clustering methods. But clustering is known to be an NP-Hard problem to find the optimal clustering under any non-trivial loss function (Aloise *et al.* 2009). This is especially true as the number of clusters (or multiplexed samples) increases (Inaba *et al.* 1994, Mahajan *et al.* 2012) with the historical solution to overcome local optima of doing multiple random restarts. It has been shown the number of clusters is in the exponent of the number of random restarts needed to even probabilistically find the global optima. More recently, the kmeans++ (Arthur and Vassilvitskii 2006) cluster initialization strategy has provided a simple but strong heuristic method for finding initial values of cluster centers that are much more likely to converge to the global optimal in the cluster optimization process. Because kmeans++ uses individual data points as initial cluster centers, and new cluster centers are chosen randomly weighted on the squared distance to the closest existing cluster center, this poses three problems when dealing with sparse data.

1. It may be unclear what value to give the cluster center in dimensions for which the data point has no data.
2. You can only judge the distance of a data point to the other cluster centers based on the dimensions for which it has data.
3. Because not every cell expresses every gene, the full gene is not covered by reads from each cell, and the experimental yield is not perfect, scRNA-seq data has a sparsity of roughly 5% (only 5% of the dimensions have data for each cell).

For these reasons, kmeans++ is not suitable for scRNA-seq data and other very sparse data types.

An alternative option would be to do a dimensionality reduction step prior to clustering. The sparse and binomial nature of this data poses some problems with this, but in a recent paper, SNPManifold utilizes a binomial variational autoencoder to learn a lower dimensional latent space of the SNP data. This is very promising work, but has not been validated on larger numbers of donors (Chung and Huang 2025). Here we present multiple algorithmic improvements for clustering scRNA-seq data by genotype. Previously, we showed that souporcell could cluster up to ∼21 individuals with very high-quality data, but we did not recommend designing experiments with >16 individuals. With these improvements, we demonstrate robust genotype-based clustering for up to 64 individuals.

## 2 Materials and methods

Souporcell3 introduces several key improvements over its predecessor souporcell (Heaton *et al.* 2020), enabling robust clustering of up to 64 donor samples. The clustering process begins with an 10× merge cluster center initialization strategy, where the algorithm generates a large number of preliminary cluster centers randomly, 10 times the expected number of donors, *k*. Rather than using these centers directly for final clustering, the algorithm performs a merging step at first, combining those that are highly similar in genotype space, typically differing by only a few informative alleles, into a more concise and representative set. This is done using a distance metric, the sum of squared differences between randomly assigned cluster center values, weighted by the number of alleles at each variant position. The two closest cluster centers are then identified and merged (by calculating the mean values for each variant position and using that as the new cluster center). This process is repeated iteratively until the desired k cluster centers are reached. This has the effect of cluster center initializations being distant from one another which reduce the chances of splitting one true cluster into two clusters (similar to kmeans++) (Arthur and Vassilvitskii 2006) (See Section 3.4, available as supplementary data at *Bioinformatics* online for a comparison of different cluster center initializing methods).

For the main clustering task, souporcell3 utilizes the K-Harmonic Means (KHM) algorithm. KHM is particularly effective in scenarios where poor cluster initialization can adversely affect outcomes, as it reduces sensitivity to initial conditions and enhances convergence to optimal solutions (Zhang *et al.* 1999, Zhang 2001, Güngör and Ünler 2007, 2008). The souporcell3 uses the KHM loss function with the negative binomial likelihood pmf from the original souporcell as the distance metric. (More details in the Section 4.1, available as supplementary data at *Bioinformatics* online). Further, souporcell3 uses a deterministic annealing variant similar to that of souporcell; however, souporcell3 applies it to KHM with a refined temperature constant. The temperature parameter starts high and gradually decreases, allowing the algorithm to explore globally optimal clusterings. (See Section 4.3, available as supplementary data at *Bioinformatics* online for a comparison of clustering methods with and without deterministic annealing).

After the initial clustering run, souporcell3 evaluates the quality of each cluster using both the number of cells assigned to the cluster and the associated loss value. Clusters that are identified as outliers, clusters deemed suboptimal, are reinitialized using the 10× merge cluster initialization strategy. Ten times the number of outlier clusters are initialized and then merged to generate well-separated cluster centers equal in number to the original outliers. This process allows the algorithm to improve clustering in the next iteration. At the same time, high-quality clusters are randomly selected from the remaining set, after excluding the outliers, and are locked to preserve their values during subsequent iterations. This strategy is inspired by node freezing and dropout in machine learning, which are used to reduce overfitting(Srivastava *et al.* 2014, Liu *et al.* 2021). This iterative refinement ensures accurate donor assignment and enhances the overall robustness of the clustering process (See Section 5, available as supplementary data at *Bioinformatics* online for more details).

## 3 Results

**Figure 1:**
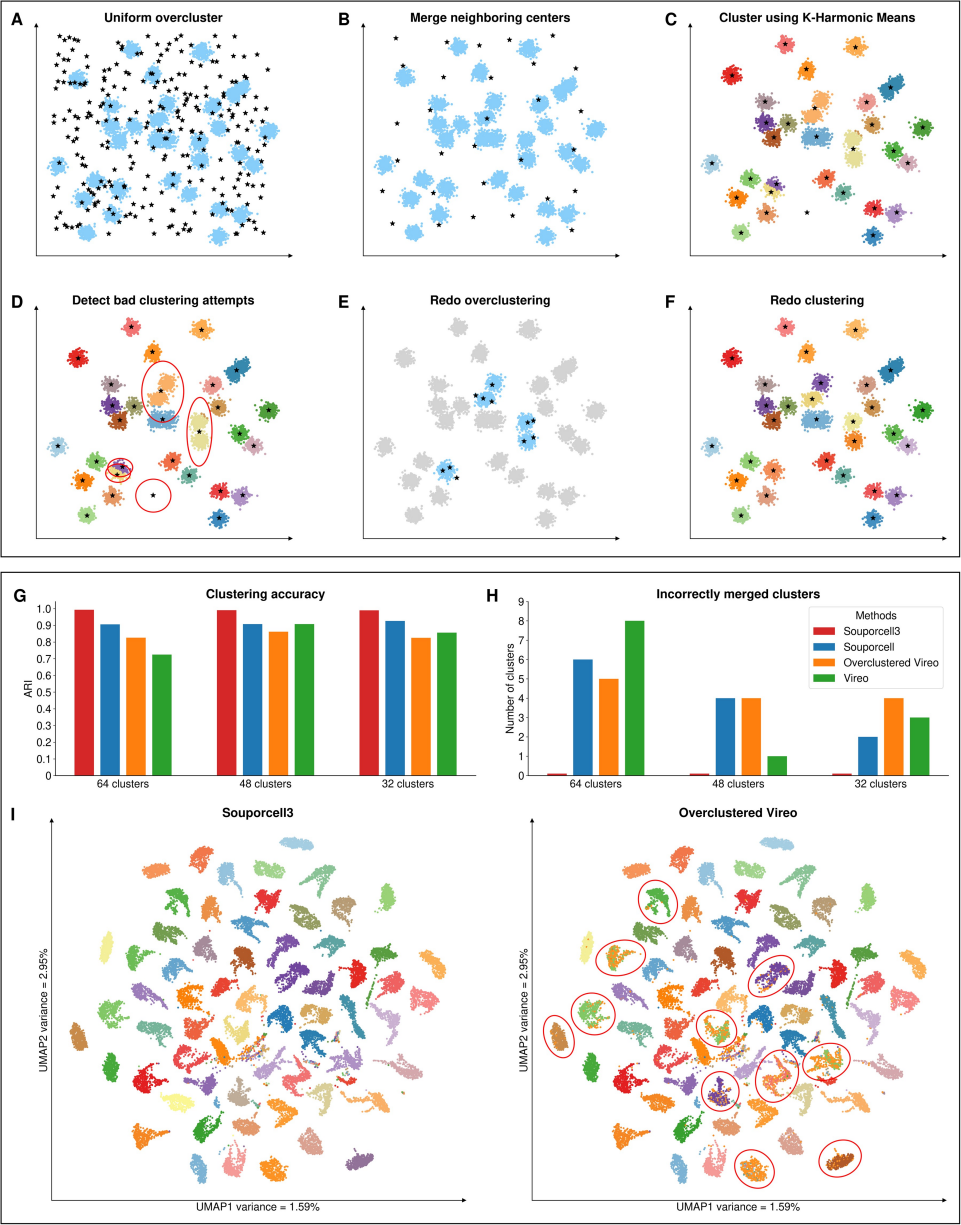
(A–F) Method overview. (A) Randomly initialize with *k* × 10 clusters to ensure broad coverage of the genotype space. (B) Merge nearby cluster centers using a distance metric defined as the sum of allele fraction differences, weighted by allele counts, until *k* clusters remain. (C) Clustering using K-Harmonic Means (KHM) to ensure enhanced convergence and reduced sensitivity to initialization. (D) Identify low- and high-quality clusters based on the number of assigned cells and the loss value per cluster. (E) Reinitialize low-quality clusters. (F) Rerun KHM on the refined set while locking the high-quality clusters. (G–I) Results: (G) ARI values for vireo, overclustered vireo, souporcell, and souporcell3 for 10% doublet, 64-donor dataset. (H) Number of incorrectly merged clusters for the same methods and dataset. (I) Cluster maps using UMAPs for the *x* and *y* axes, showing the result of 10% doublet, 64-donor dataset after running souporcell3 (left) and overclustered vireo (right) with poor clustering results highlighted with circles.

To assess the demultiplexing accuracy and robustness of our improved souporcell method, we constructed benchmark datasets by integrating single-cell RNA-seq data from four different sources (see Section 1.1, available as supplementary data at *Bioinformatics* online for dataset composition and preprocessing details). These datasets were specifically designed to test performance under increasing levels of complexity, including doublet rates of 0%, 5%, and 10%. The results shown are using the 10% doublet rate 64-donor dataset, which is slightly higher than the expected 8% for a sample containing ∼20 000 cells (Ortolano 2024) (Fig. 1B and C).

We benchmarked our method against three state-of-the-art genotype-based demultiplexing approaches: vireo (using its default configuration), overclustered vireo (where we specified the pre-cluster donors to *k* × 10 to match the number of clusters in our 10× merge), and the original souporcell (run with its default settings), while souporcell3 used the method described above.

The UMAP plots in (Fig. 1I) display cells colored by their ground truth donor identity and project the high-dimensional clustering into two dimensions along the UMAP1 and UMAP2 axes, capturing the overall data structure. In the left plot, representing souporcell3 clustering, clusters are well-separated across the space. Even in densely packed regions, such as the center, the clusters remain clearly partitioned, with only a few outliers, highlighting souporcell3’s ability to resolve tightly grouped but distinct donor profiles. By contrast, the overclustered vireo clustering shown in the right plot, exhibits several artifacts, including incorrectly merged clusters where cells from a single donor are split into multiple clusters (circled), indicating reduced clustering accuracy in complex settings.

Clustering outcomes were evaluated using two primary metrics: the presence of incorrectly merged clusters after convergence (Fig. 1H), and clustering accuracy as measured by the ARI (Rand 1971, Hubert and Arabie 1985, Vinh *et al.* 2010) (Fig. 1G) (See Section 2.1, available as supplementary data at *Bioinformatics* online for explanation of the metrics and Section 6.1, available as supplementary data at *Bioinformatics* online for results on other datasets). Incorrectly merged cluster analysis (Fig. 1H) shows that in high donor counts, our enhanced souporcell3 method produced no incorrectly merged clusters, in contrast to the comparison methods, which generated multiple such artifacts, particularly at higher donor counts. ARI analysis (Fig. 1G) further confirms the accuracy of our method, with consistently higher values across all conditions compared to the alternative methods. These results indicate the improvements introduced in our pipeline not only eliminate incorrectly merged clusters but also significantly enhance clustering fidelity.

Overclustered vireo outperformed vireo with its default setting, highlighting that initializing cluster centers with additional pre-clustered donors yields better performance in high-donor scenarios. Souporcell, utilizing its expectation–maximization algorithm with random cluster center initialization, performed better than both versions of vireo which uses variational Bayesian inference. Souporcell3 improves upon this by incorporating a multi-stage clustering pipeline that combines 10× merge initialization, K-harmonic means, and iterative cluster refinement with selective cluster locking. This accuracy gain remains robust even as data complexity and size increase, indicating that our method is well-suited for large-scale single-cell studies involving pooled donors.

In addition to these in silico mixtures, we further validated souporcell3 with three true biological mixtures from SNPManifold (Jerber *et al.* 2021, Chung and Huang 2025) to test souporcell3’s performance on unbalanced datasets in which some clusters have far fewer cells than the average cluster. We tested souporcell3 on datasets with 18, 24, and 19 individuals respectively with minority clusters of 0.84%, 1.4%, and 1.13%, respectively, with a perfect adjusted rand index of 1 for cells labeled as individual donors by demuxlet (See Section 6.2, available as supplementary data at *Bioinformatics* online).

## 4 Conclusion

Our evaluation of genotype-based demultiplexing using a complex dataset demonstrates that the improved souporcell3 approach consistently enhances clustering performance. The method scales effectively to 64 donors while maintaining high accuracy under varying levels of doublets and across different dataset complexities. By combining 10× merge initialization, K-Harmonic Means clustering, and iterative refinement with selective cluster locking, our method eliminates common artifacts such as empty or incorrectly merged clusters and achieves higher concordance with ground truth donor labels. These enhancements highlight the advantages of robust initialization and adaptive cluster correction strategies in high-throughput demultiplexing. Future work will continue to refine the method in the context of different data types such as scATACseq and long read scRNA-seq with even larger sample sizes and increasingly complex single-cell datasets, ensuring accurate and scalable demultiplexing for population-scale single-cell studies.

## Supplementary Material

btag117_Supplementary_Data

## Data avaliability

Souporcell3 source code and documentation are released on GitHub: https://github.com/wheaton5/souporcell and Zenado with doi: https://doi.org/10.5281/zenodo.17665558. The single-cell RNA-seq datasets used and analyzed in this study are publicly available from the Human Cell Atlas under the following project accessions: 3089d311-f9ed-44dd-bb10-397059bad4dc, cc95ff89-2e68-4a08-a234-480eca21ce79, and fcaa53cd-ba57-4bfe-af9c-eaa958f95c1a. European Nucleotide Archive (ENA) data used are available under sample accessions ERS2630502–ERS2630507 corresponding to the cell lines euts, nufh, babz, oaqd, and ieki. The unbalanced datasets under sample accession SAMEA6833385, SAMEA6833405, and SAMEA6833432.
