# Supplementary material for: Souporcell3: robust demultiplexing for high-donor single-cell RNA-seq datasets
Source: Bioinformatics. 2026 Mar 10;42(3):btag117. doi: 10.1093/bioinformatics/btag117 (PMC13012599; doi:10.1093/bioinformatics/btag117)
Supplement: btag117_Supplementary_Data [file btag117_supplementary_data.pdf]

SUPPLEMENTARY

1. Datasets

To evaluate our tool under unbalanced conditions, we used three high-donor datasets containing 18–24 donors that were pooled and sequenced (Jerber *et al.* 2021). To assess performance with larger donor counts (~64 donors), we obtained individual datasets from established publications (Cillo et al. 2020, Jardine et al. 2021) and synthetically merged them.

1.1 Balanced Synthetically Merged Datasets

1.1.1 Data used

We utilized four datasets from the Human Cell Atlas and European Nucleotide Archive, each comprising human-derived samples and generated using the 10X Genomics 3' chemistry (v1 and v2) to generate the high donor synthetically merged balanced dataset. All samples were derived from Homo sapiens, and the data collectively span a wide range of immune and developmental contexts, offering a diverse foundation for evaluating demultiplexing performance across donor pools and biological conditions.

Source 1

Publication: Immune Landscape of Viral- and Carcinogen-Driven Head and Neck Cancer (Cillo et al. 2020)

Donors: 37

Cell count: 126.0k

Dataset: (Human Cell Atlas 2020a)

Source 2

Donors: 28

Cell count: 1.5M

Dataset: (Human Cell Atlas 2020b)

Source 3

Publication: Mapping the developing human immune system across organs (Jardine et al. 2021)

Donors: 30

Cell count: 922.3k

Dataset: (Human Cell Atlas 2021)

Source 4

Donors: 5

Cell count: 50k

Dataset: (ENA 2020a)

Collectively, these datasets span 100 unique donors and over 2.5 million single-cell transcriptomes, providing a rich and diverse foundation for evaluating and benchmarking single-cell demultiplexing methods in real-world, heterogeneous biological contexts.

1.1.2 Donor allocation

Out of the 100 remaining donors, we filtered out samples with corrupted data, insufficient read depth, and redundant samples from the same individual collected across different organs. After applying these quality control and deduplication steps, we curated a high-quality balanced dataset comprising 64 distinct donors for high donor clustering evaluation. The table below summarizes the composition of these 64 donors and their respective sources.

Table 1. Shows the source and the original ID of the modified ID used in the CB tag of our dataset for differentiating donors.

Modified ID	Original ID	Source	Modified ID	Original ID	Source
0	euts	ENA 2020a	35	CB6	Human Cell Atlas 2020b
1	BL1	Human Cell Atlas 2020b	36	ieki	ENA 2020a
2	CB2	Human Cell Atlas 2020b	37	F38	Human Cell Atlas 2021
3	HNSCC_9	Human Cell Atlas 2020a	38	F45	Human Cell Atlas 2021
4	HNSCC_3	Human Cell Atlas 2020a	39	F32	Human Cell Atlas 2021
6	HD_5	Human Cell Atlas 2020a	41	BL7	Human Cell Atlas 2020b
7	HNSCC_20	Human Cell Atlas 2020a	42	CB4	Human Cell Atlas 2020b
8	HD_tonsil2	Human Cell Atlas 2020a	43	F30	Human Cell Atlas 2021
9	BM8	Human Cell Atlas 2020b	44	HNSCC_14	Human Cell Atlas 2020a
10	HNSCC_18	Human Cell Atlas 2020a	45	BM4	Human Cell Atlas 2020b
11	BM2	Human Cell Atlas 2020b	46	CB11	Human Cell Atlas 2020b
12	BM5	Human Cell Atlas 2020b	47	F23	Human Cell Atlas 2021
14	CB10	Human Cell Atlas 2020b	48	F29	Human Cell Atlas 2021
15	HD_2	Human Cell Atlas 2020a	49	HD_3	Human Cell Atlas 2020a
16	CB5	Human Cell Atlas 2020b	50	HNSCC_21	Human Cell Atlas 2020a

17	BL6	Human Cell Atlas 2020b	51	HD_4	Human Cell Atlas 2021
18	nufh	ENA 2020a	52	BM3	Human Cell Atlas 2020b
19	BL8	Human Cell Atlas 2020b	53	HNSCC_13	Human Cell Atlas 2020a
20	CB1	Human Cell Atlas 2020b	54	HNSCC_19	Human Cell Atlas 2020a
21	BL2	Human Cell Atlas 2020b	55	F61	Human Cell Atlas 2021
22	F51	Human Cell Atlas 2021	56	CB9	Human Cell Atlas 2020b
23	HNSCC_23	Human Cell Atlas 2020a	57	HNSCC_2	Human Cell Atlas 2020a
25	BM1	Human Cell Atlas 2020b	58	CB3	Human Cell Atlas 2020b
26	HNSCC_11	Human Cell Atlas 2020a	59	CB7	Human Cell Atlas 2020b
27	HNSCC_16	Human Cell Atlas 2020a	60	BL4	Human Cell Atlas 2020b
28	BM6	Human Cell Atlas 2020b	61	HNSCC_6	Human Cell Atlas 2020a
29	F19	Human Cell Atlas 2021	62	oaqd	ENA 2020a
30	HNSCC_24	Human Cell Atlas 2020a	63	BM7	Human Cell Atlas 2020b
31	HD_1	Human Cell Atlas 2020a	64	CB12	Human Cell Atlas 2020b
32	F21	Human Cell Atlas 2021	66	HNSCC_22	Human Cell Atlas 2020a
33	HNSCC_7	Human Cell Atlas 2020a	67	babz	ENA 2020a
34	BL5	Human Cell Atlas 2020b	69	BL3	Human Cell Atlas 2020b

1.1.3 Dataset generation

To make a number of balanced datasets we generated subsets tailored for benchmarking by extracting the required number of cells for each experimental scenario. Specifically, we constructed datasets with controlled doublet rates of approximately 0%, 5%, and 10% and donor numbers. Each subset was limited to a total of around 10,000~30,000 cells, aligning with the practical upper limit for many current single-cell RNA-sequencing (scRNA-seq) experiments using 10X Genomics platforms (10X Genomics 2020). This cell count threshold reflects a balance between throughput and data quality, as higher cell input often increases the likelihood of doublet formation and misclassification errors. Published reports suggest that, at this scale, the expected doublet rate for standard 10X Chromium 3' chemistry ranges between 5% and 10% when targeting ~20,000 cells (10X Genomics 2020; Zheng et al. 2017; Wolock et al. 2019; McGinnis et al. 2019).

Table 2. Shows the composition of different balanced datasets used in evaluation.

Type	Donors	Total cell count	Cells per donor	Doublet count	Doublet percentage
No doublets	4	9300	2325	0	0
	8	10000	1250	0	0

	16	12800	800	0	0
	32	12800	400	0	0
	48	19200	400	0	0
	64	25600	400	0	0
5 percent doublet	4	7123	1781	373	5.23655763
	8	8303	1038	428	5.154763339
	16	11200	700	578	5.160714286
	32	11777	368	617	5.239025219
	48	18239	380	959	5.257963704
	64	20061	313	1053	5.248990579
10 percent doublet	4	6740	1685	740	10.97922849
	8	8120	1015	888	10.93596059
	16	10799	675	1199	11.1028799
	32	11159	349	1239	11.10314544
	48	16917	352	1877	11.09534787
	64	22679	354	2519	11.10719168

1.2 Unbalanced Experimentally Merged Dataset

1.2.1 Data used

We utilized well-established imbalanced datasets (Jerber *et al.* 2021), previously used in multiple publications (Chung and Huang 2025), comprising 18–24 donors, to demonstrate the robustness of our method in a real experimental scenario. All three datasets were obtained from the European Nucleotide Archive (ENA) and consist of human-derived samples generated using the 10x Genomics 3′ chemistry (v1 and v2).

<u>Source 5 (unbalanced 1)</u>

Pool: 4

Donors: 18

Cell count: 8k

Dataset: (ENA 2020b)

<u>Source 6 (unbalanced 2)</u>

Pool: 6

Donors: 24

Cell count: 6k

Dataset: (ENA 2020c)

Source 7 (unbalanced 3)

Pool: 8

Donors: 19

Cell count: 8k

Dataset: (ENA 2020d)

1.2.2 Donor allocation

The table below summarizes the composition of these unbalanced datasets and the corresponding cell counts per donor. The ground-truth donor assignments were determined using Demuxlet (Xu *et al.* 2019), as reported in the dataset's original publication (Jerber *et al.* 2021).

Table 3. Shows the composition of different unbalanced datasets used in evaluation.

Pool 4 (Unbalanced 1)			Pool 6 (Unbalanced 2)			Pool 8 (Unbalanced 3)		
Modified ID	Original ID	Cells	Modified ID	Original ID	Cells	Modified ID	Original ID	Cells
0	HPSI0115i-aion_2	324	0	HPSI0114i-wegi_1	484	0	HPSI0115i-aoxv_1	855
1	HPSI0115i-bimq_4	271	1	HPSI0115i-zihe_1	153	1	HPSI0215i-romx_1	621
2	HPSI0115i-boqx_2	73	2	HPSI0214i-heth_1	171	2	HPSI0215i-zett_5	667
3	HPSI0115i-gifk_1	83	3	HPSI0215i-hipn_1	335	3	HPSI0314i-xugn_2	391
4	HPSI0214i-feec_2	375	4	HPSI0215i-yoch_6	160	4	HPSI0414i-oaqd_2	339
5	HPSI0214i-giju_3	492	5	HPSI0314i-qaqx_1	142	5	HPSI0414i-uawq_2	376
6	HPSI0215i-deyz_2	711	6	HPSI0314i-uict_1	169	6	HPSI0414i-xojn_3	527
7	HPSI0215i-fawm_2	698	7	HPSI0314i-xagu_1	89	7	HPSI0514i-oekw_2	272
8	HPSI0314i-cuhk_2	471	8	HPSI0414i-mita_1	500	8	HPSI0514i-wiii_3	223
9	HPSI0314i-sojd_3	221	9	HPSI0514i-qihv_1	244	9	HPSI0614i-miaj_4	770
10	HPSI0414i-ceik_1	192	10	HPSI0514i-suul_1	228	10	HPSI0614i-qunz_2	93

11	HPSI0514i-fiaj_1	896	11	HPSI0514i-tert_1	287	11	HPSI0715i-aowh_2	515
12	HPSI0514i-lako_1	335	12	HPSI0614i-voce_1	283	12	HPSI0715i-zaie_5	409
13	HPSI0714i-burb_1	402	13	HPSI0714i-oatm_1	152	13	HPSI0914i-ouvb_2	343
14	HPSI0814i-bokz_6	147	14	HPSI0914i-laey_6	207	14	HPSI0914i-zerv_7	195
15	HPSI1014i-bilx_1	66	15	HPSI1013i-hiaf_1	414	15	HPSI1013i-sita_1	532
16	HPSI1014i-eesb_1	1514	16	HPSI1013i-woci_1	321	16	HPSI1014i-babz_3	442
17	HPSI1213i-hehd_1	605	17	HPSI1013i-zagm_1	100	17	HPSI1014i-kefb_1	145
			18	HPSI1014i-nosn_1	167	18	HPSI1014i-vils_1	500
			19	HPSI1014i-sehl_6	305			
			20	HPSI1014i-tuju_1	213			
			21	HPSI1114i-kuul_1	206			
			22	HPSI1114i-ualf_6	128			
			23	HPSI1213i-tolg_4	78			

2.1 Evaluation metrics: Adjusted Rand Index (ARI) and Incorrectly Merged Clusters (IMC)

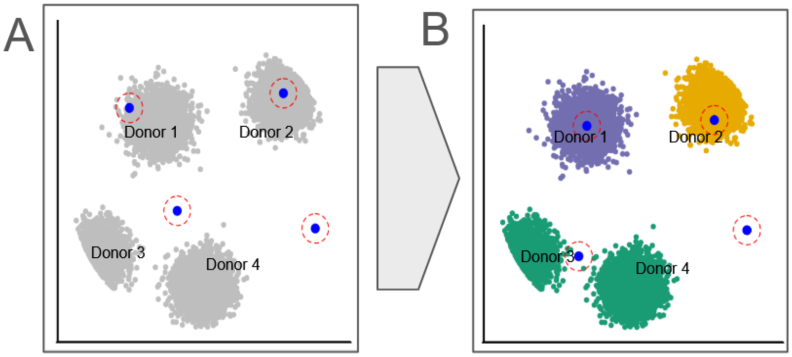

Figure 1. A. Before clustering, cluster centers are initialized **B.** After the clustering method converges, an empty cluster center is present (not assigned to any cells) and one cluster center is assigned to cells from two donors.

Throughout the main text and supplementary materials, we evaluate clustering performance using two key metrics: the Adjusted Rand Index, or ARI, and the number of Incorrectly Merged Clusters, or IMC. The Rand Index is a standard and widely used metric in clustering analysis; it measures the similarity between two data clusterings (Rand 1971). A more advanced method, known as the Adjusted Rand Index, is introduced to account for the random chance the clustering gets assigned correctly (Hubert et al. 1985). In our case, ARI is used, and we compare the clustering output of each method against the known ground truth, which is available since the datasets were generated from sources with known donor origins. The second metric, IMC, captures instances where two or more distinct ground-truth donors are incorrectly assigned to the same cluster by the method. In figure 1B, the IMC value is one, as both donor 3 and donor 4 cells are incorrectly grouped into the same cluster by the clustering algorithm.

2.2 Input / data preprocessing

Data preprocessing is performed similarly to souporcell. To prepare the data for genotype-based cell clustering, we first measure the allele information for each cell. Instead of using STAR-aligned BAM files, which can introduce false-positive variants and reference bias, we remap the reads using minimap2 to obtain more accurate alignments. Putative single-nucleotide polymorphisms (SNPs) are then called with freebayes, and allele counts per cell are obtained using vartrix, which reduces reference bias and corrects duplicate reads through unique molecular identifiers (UMIs). When available, a set of reliable common variants can be used instead of freebayes-derived candidate variants. The alternate/reference read depth at each variant locus is used to determine whether the locus is included in the clustering task. A default threshold of 4 is set in Souporcell3, which can be adjusted by the user. The allele frequency at each passing locus is used for the binomial loss calculation and, in turn, for the clustering task.

3. Cluster Initialization

3.1 *k*-means++ initialization

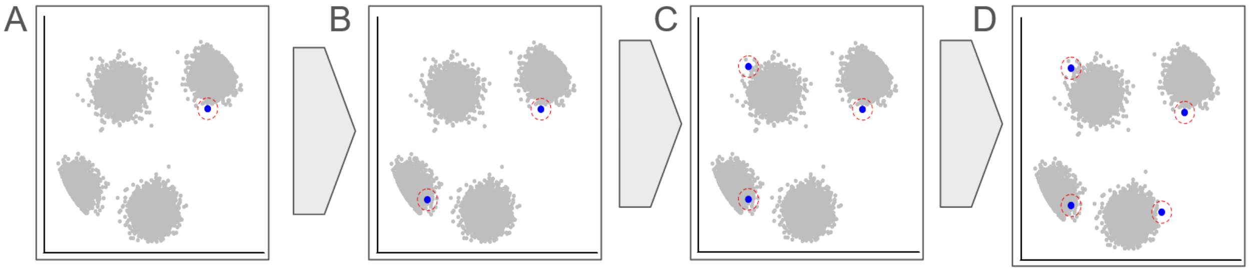

Figure 2. A. Randomly select a cell to be the first cluster center **B, C, D.** Iteratively select cells as cluster centers based on their squared distance to the closest existing cluster center.

Almost all commonly used clustering algorithms implicitly assume the data is dense and continuous, which ensures a relatively smooth cost function landscape. In contrast, genetic data is both sparse and discrete, with each dimension typically limited to three possible values. This sparsity poses significant challenges for greedy clustering algorithms like *k*-means, which are prone to getting trapped in local minima. A standard remedy is to perform multiple random restarts; however, the number of restarts needed to reliably approach the global optimum increases exponentially with the number of clusters *k* (Aloise et al. 2009, Inaba et al. 1994). To address this, the *k*-means++ initialization method was proposed (Arthur & Vassilvitskii 2007), offering a simple, parameter-free, and generally robust strategy. Despite its widespread adoption, *k*-means++ still struggles in sparse settings, where limited shared information between data points across dimensions undermines the effectiveness of its distance-based seeding.

In *k*-means++, the initial cluster center is selected randomly from the dataset. A distance metric is then used iteratively to determine the probability of selecting a new cluster center, based on the weighted squared distance of each datapoint from its closest existing cluster center. For this metric, we used the beta-binomial distance, as it accounts for genotype positions where no data is present.

3.2 Overclustering initialization

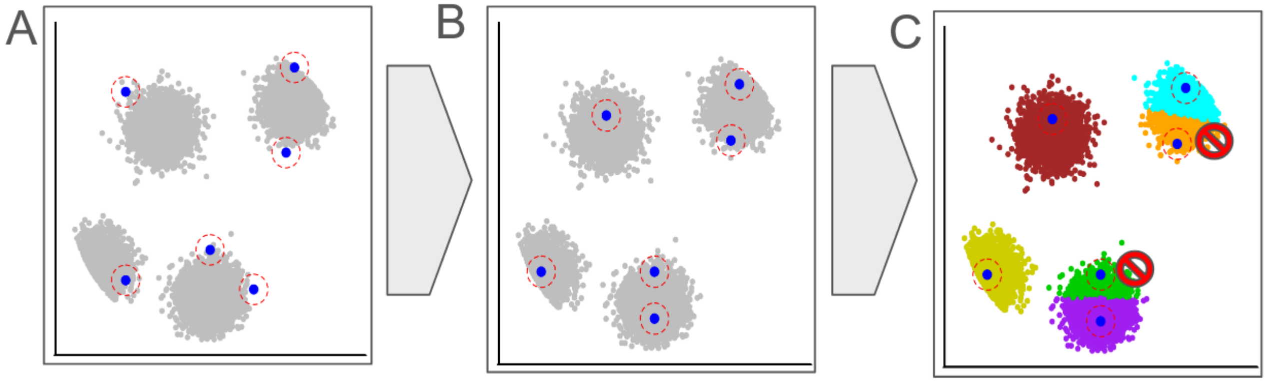

Figure 3. A. Selecting $k + \sqrt{k}$ random cells as cluster centers, where k is the desired number of clusters **B.** Clustering using the selected cluster centers **C.** Select k cluster centers with maximally associated cells.

Another strategy aimed at improving cluster initialization is overclustering. It is suited for challenging genetic datasets, such as those that are sparse and discrete. Traditional clustering algorithms often struggle in these contexts due to poor initial seeding, which can lead to convergence on suboptimal solutions. By temporarily increasing the number of clusters beyond the desired value, overclustering aims to better capture the underlying structure of the data, especially since the effective number of clusters can be higher than expected due to the presence of doublets. This strategy is employed by Vireo and SC3 (Huang et al. 2019, Kiselev et al. 2017) to enhance cluster initialization.

We begin clustering with $k + \sqrt{k}$ clusters instead of the target k. After performing the clustering step, we evaluate the resulting clusters based on the number of cells assigned to each. The k clusters with the highest cell counts are then selected to form the final set of cluster centers, similar to Vireo. This selection criterion ensures that the most representative and well-supported clusters are retained, while smaller, potentially spurious clusters are discarded. However, this method can be prone to errors in high-donor-count scenarios, where the genotype space is limited and substantial overlap between donors exists. In such cases, selecting clusters solely based on cell count may result in incorrectly merged donors, where one cluster gets assigned cells of multiple donors.

3.3 10× merging initialization

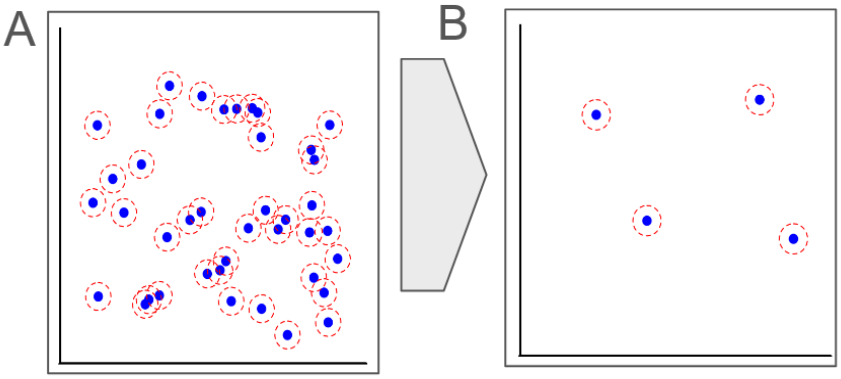

Figure 4. A. Randomly initializing 10× the number of *k* clusters required **B.** Merging clusters in close proximity with each other until required cluster size reached.

10× merging is the primary initialization strategy employed by Souporcell3, where the algorithm generates a large number of preliminary cluster centers, ten times the expected number of donors. Rather than using these centers directly for final clustering, the algorithm performs a merging step at first, combining those that are highly similar in genotype space, typically differing by only a few informative alleles, into a more concise and representative set. Using a distance metric, the sum of squared differences between randomly assigned cluster center values, weighted by the number of alleles at each variant position, we identify the two closest cluster centers and merge them (by calculating the mean values for each variant position and using that as the new cluster center). This process is repeated iteratively until the desired *k* cluster centers are reached.

10× merging reduces redundancy while preserving essential genotype distinctions, ensuring that the actual clustering step begins with informative and well-spread cluster centers. This is particularly helpful in scenarios where multiple overlapping donor cells occupy a dense genotype space, as widely separated cluster centers are unlikely to claim cells from multiple donors at the outset.

3.4 Cluster initialization evaluation

To evaluate the different cluster initialization methods, we tested them on our 64-donor dataset without doublets. We also included an optimal cluster initialization blueprint, where clusters were initialized using ground truth donor information, in order to assess whether perfect initialization alone could give perfect clustering or if additional refinement steps were still necessary in high-donor scenarios.

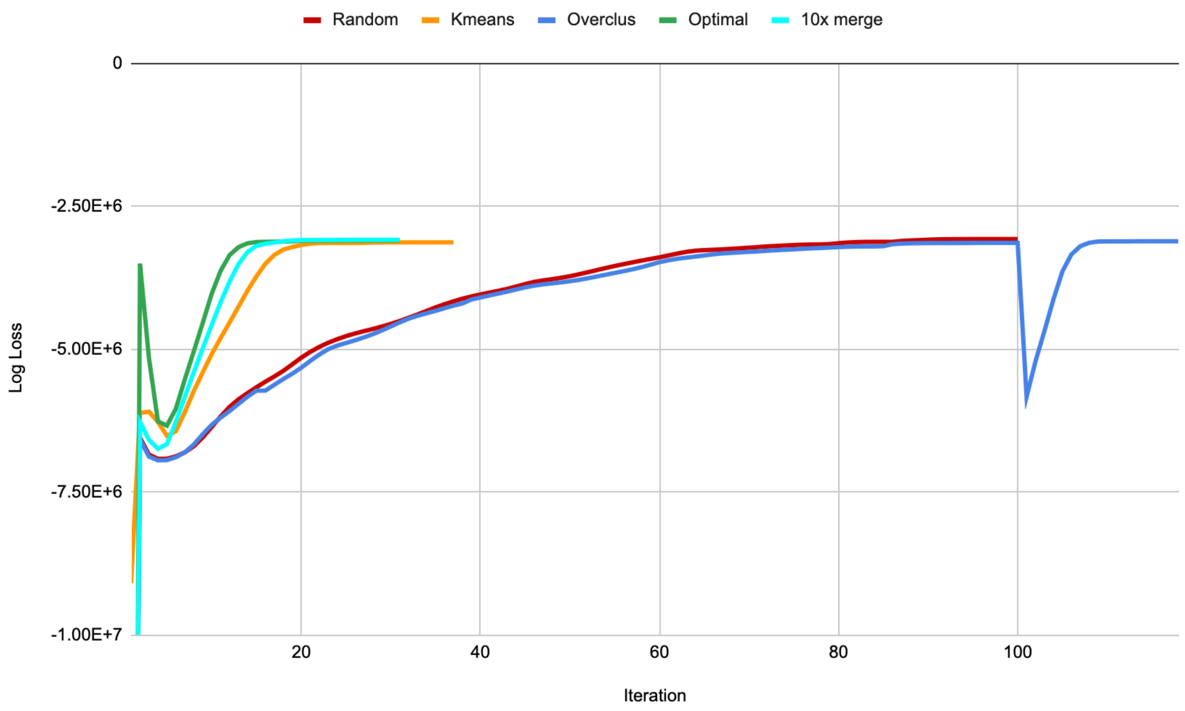

Figure 5. Log loss vs. iterations for EM with 5 different initialization methods: random, kmeans, overclustering, optimal, and 10× merge. A no-doublet balanced dataset with 64 donors was used for the experiment.

The log loss, number of iterations relationship for a no-doublet dataset of 64 donors is shown in the figure. Expectation-maximization (EM) with Optimal cluster initialization started off with the lowest log loss but eventually reached the same log loss as kmeans and 10× merge, showing the same pattern. Both random and overclustering methods took more iterations to converge, but overclustering ran for more iterations after removing the extra $\sqrt{k}$ clusters (100+ iterations) and converged to the same log loss as Random.

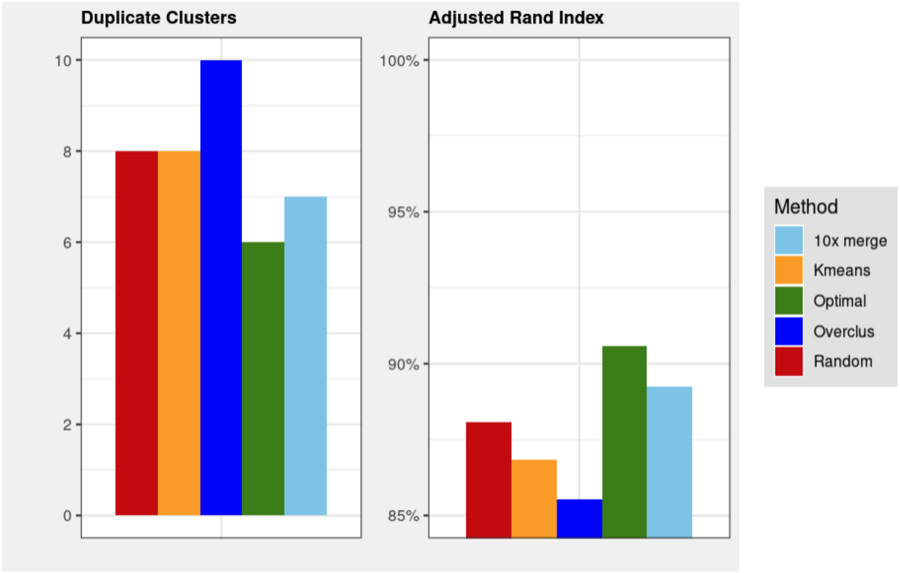

Figure 6. The right side shows the ARI values for an EM run with different cluster initialization methods. The left side illustrates the duplicate assignments (incorrectly merged clusters) present for the methods. The dataset used for figure 5 was also utilized for this evaluation.

The bar charts display two key clustering metrics: incorrectly merged clusters (or IMC) and ARI, across five initialization methods: 10× merge, kmeans, optimal, overclustering, and random. As shown on the left, the number of incorrectly merged clusters is lowest for the optimal initialization method, which is expected since the cluster centers were initialized with ground truth values. overclustering and random initializations resulted in the highest number of incorrectly merged clusters, with overclustering making 10. Among the practical initialization methods, 10× merge performed the best, producing around 7 incorrectly merged clusters. On the right, the ARI values reinforce this trend, with the optimal method achieving the highest accuracy (> 90%), while their overclustering and random counterparts show lower performance (< 88%).

Interestingly, when using the optimal initialization, which uses ground truth information to set the cluster centers, the ARI value improved only marginally by 1.3%, and incorrectly merged cluster assignments were reduced by just one, when compared to 10× merge. This is unexpected, as perfect clustering is theoretically possible under such conditions, where no further updates to the cluster centers should be needed. However, even after convergence, incorrectly merged cluster assignments still occurred. This may be due to the large number of donors involved, where cells are densely packed in the genotype space. As the optimization progresses, log loss updates may

inadvertently shift a cluster center toward a dense region, causing it to claim ownership of cells from neighboring donors with similar genotypes, thus introducing incorrectly merged clusters.

4. Clustering

We showed that the high-donor clustering problem cannot be solved alone with cluster initialization; we try exploring the KHM clustering method to determine whether any improvements can be made.

4.1 K-Harmonic Means clustering

Soft clustering methods, such as Expectation-Maximization (EM) applied to Gaussian Mixture Models (GMMs), have been widely used due to their ability to recover more optimal clusterings than hard clustering methods like Lloyd's algorithm for k-means (Peizhuang 1983). However, these methods are more prone to converging to local optima if the cluster initialization is not done correctly. To address this, we adopt K-Harmonic Means (KHM) (Zhang, Hsu & Dayal 1999, Güngör & Ünler 2007, Güngör & Ünler 2008), a soft clustering objective that computes the contribution of each data point to all cluster centers based on inverse distances. Similar to souporcell, cluster centers in souporcell3 are represented by allele fraction values for each variant locus. souporcell3 uses the negative binomial likelihood pmf of allele counts at each cluster center's allele fraction as the distance metric for the KHM loss function, calculated according to the original formulation (Zhang, Hsu & Dayal, 1999).

Definitions:

• K: the number of genotype clusters to be fixed at the outset. Lower-case k is used for indexing and referring to a specific cluster.

• C: the number of cells. Lower-case c is used for indexing and referring to a specific cell barcode. This barcode could have 0, 1 or more cells. It is important for some assumptions in this model that the majority of barcodes contain a single cell.

• L: the number of variant loci. Lower-case l is used to index and refer to a specific locus. We assumed only biallelic variants. L_c is a list of loci with observed data in cell c.

• A: allele counts. $A_{l,c}$ is a tuple of size 2 with the first number representing the number of reference alleles and the second number representing the number of alternate alleles seen at locus l in cell c.

• $\phi_{k,l}$: mixture parameter for allele fractions of cluster k at locus l. This is a real number representing the fraction of reference alleles in this cluster at this locus. We expect this to be near 1.0 (homozygous reference), 0.5 (heterozygous) or 0.0 (homozygous alternate) but it can be skewed from these values by noise, doublets or ambient RNA.

Distance metric:

$$d_{c,k} = -\prod_{l \in L_c} \binom{A_{l,c,0} + A_{l,c,1}}{A_{l,c,1}} \phi_{k,l}^{A_{l,c,1}} \left(1 - \phi_{k,l}\right)^{A_{l,c,0}} \tag{1}$$

KHM update:

$$m_{c,k} = \frac{1}{d_{c,k}^3 \left(\sum_{i \in K} \frac{1}{d_{c,i}^2}\right)^2} \tag{2}$$

$$\phi'_{k,l} = \frac{\sum_{c \in C} (A_{l,c,0}) m_{c,k}}{\sum_{c \in C} (A_{l,c,0} + A_{l,c,1}) m_{c,k}} \tag{3}$$

KHM loss function:

$$\mathcal{L} = \prod_{c \in C} \frac{K}{\sum_{k \in K} d_{c,k}^2} \tag{4}$$

This formulation allows every point to influence all centers during each iteration, leading to more robust convergence and improved clustering outcomes. KHM has been shown to outperform traditional k-means in both optimality and resilience to poor or adversarial initializations. To further adapt KHM to high-dimensional genotype space, we employ a generalized variant, KHMp (Zhang 2001), which uses Lp-norm normalization to address symmetry issues known to affect clustering performance in such contexts. This introduces a tunable hyperparameter, p, that controls the influence of distance magnitudes. We found that setting p = 25 minimized the loss across a range of tested values, and this was used in all reported results.

4.2 Deterministic annealing

Souporcell used a deterministic annealing variant of expectation–maximization (DA_EM) to manage non-convexity via a “temperature” parameter that starts high and is gradually lowered; this approach allows the solution to more often fall into the global optimal clustering. Similarly, souporcell3 employs a deterministic annealing-enhanced KHM (DA_KHM), combining the robustness of KHM with the annealing strategy: The temperature parameter is slightly adjusted using a temperature constant to account for the difference in convergence speed between the two clustering algorithms. When the temperature reaches 1.0, the loss function effectively becomes the KHM loss function. We compare the deterministic annealed methods and the vanilla methods below.

4.3 K-Harmonic Means evaluation

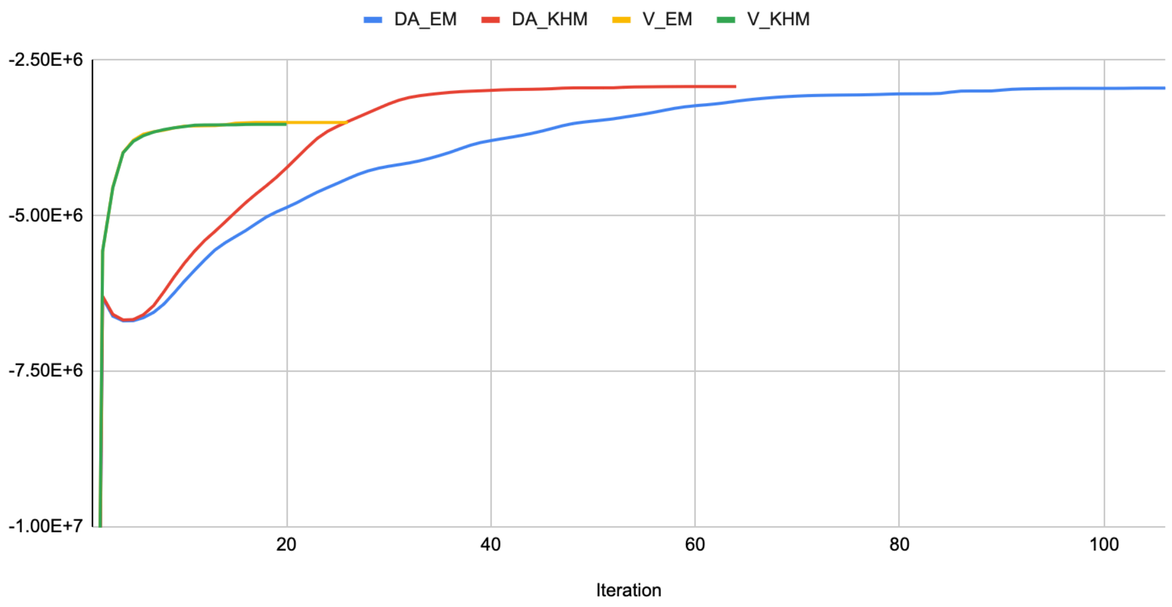

Figure 7. Log loss vs. iterations for clustering methods: DA_EM (Expectation maximization with deterministic annealing), DA_KHM (K Harmonic means with deterministic annealing), V_EM (Expectation maximization without deterministic annealing), and V_KHM (K Harmonic means without deterministic annealing). A no-doublet balanced dataset with 64 donors was used.

The log loss versus iteration for a no-doublet dataset of 64 donors is shown in the figure. EM and KHM without deterministic annealing (V_EM and V_KHM) followed a similar pattern and converged to approximately

the same log loss. Both deterministic annealing methods required more iterations to converge, with DA_EM taking about twice as many iterations as DA_KHM. DA_EM converged to a slightly worse log loss of -2,951,437.3 compared to -2,927,414.5 for DA_KHM.

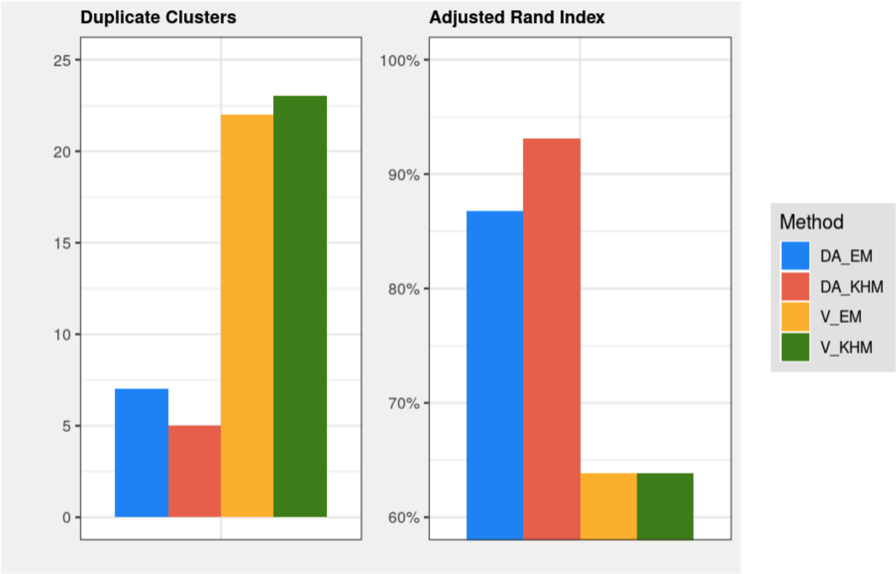

Figure 8. The right side shows the Adjusted Rand Index values for clustering methods DA_EM (Expectation maximization with deterministic annealing), DA_KHM (K Harmonic means with deterministic annealing), V_EM (Expectation maximization without deterministic annealing), and V_KHM (K Harmonic means without deterministic annealing). The left side illustrates the duplicate assignments (incorrectly merged clusters) present in the same methods. A no-doublet balanced dataset with 64 donors was used for the evaluation.

The bar plots present the performance of four clustering methods, DA_EM, DA_KHM, V_EM, and V_KHM, on a no-doublet dataset of 64 donors. The left panel shows the number of incorrectly merged clusters, and the right panel displays the Adjusted Rand Index (ARI), which reflects clustering accuracy. Among the methods, DA_KHM achieved the highest ARI, exceeding 93 percent, and also had the fewest incorrectly merged clusters, just five. DA_EM also performed well, with an ARI close to 90 percent and only seven incorrectly merged clusters. In contrast, both non-annealing variants (V_EM and V_KHM) performed significantly worse, with ARI values around 63 percent and more than 22 incorrectly merged clusters each. These results demonstrate the substantial benefit of deterministic annealing in clustering complex genotype data.

The more robust DA_KHM method outperformed DA_EM by 5% in ARI and produced 2 fewer incorrectly merged clusters. While this represents a significant improvement, perfect clustering is still not achieved, as some incorrectly merged cluster assignments remain.

5. Rerun with cluster reinitialization

5.1 Bad cluster center detection

We analyzed the cell-to-cluster assignments in the 64-donor, no-doublet dataset after algorithm convergence to investigate our hypothesis regarding incorrectly merged cluster assignments, specifically that cluster centers may incorrectly claim cells from other donors due to the constrained genotype space, and to develop a potential solution.

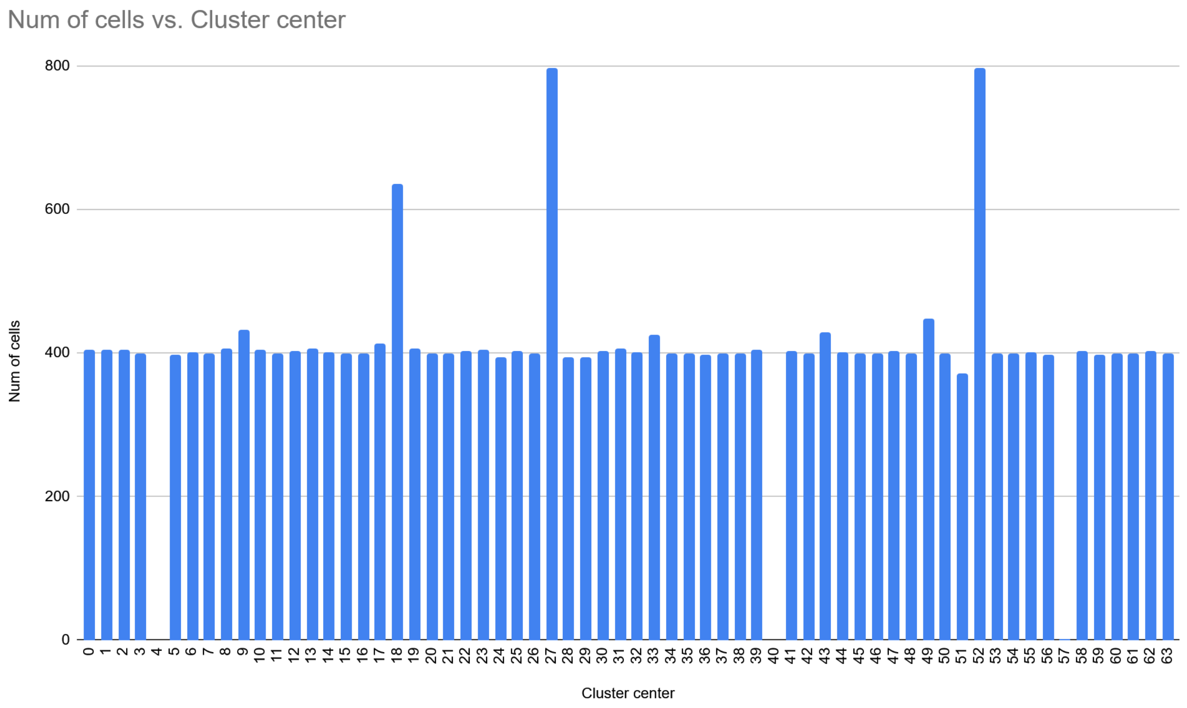

Figure 9. Number of cells assigned to each cluster center. A no-doublet balanced dataset with 64 donors was used.

Analyzing the clustering output, we found that the number of incorrectly merged clusters corresponds to empty or near-empty clusters. As shown in the figure, the cell assignments to each cluster center after convergence reveal three incorrectly merged cluster centers, which align with the presence of three empty clusters.

By computing the total log loss for each cell's assigned cluster, normalizing it by the mean total log loss, and identifying statistical outliers, we can effectively pinpoint cluster centers that are poorly or incorrectly assigned.

$$CC\ Value = \sum_{all\ cells} \frac{Log\ loss\ for\ assigned\ CC}{Mean\ log\ loss}$$

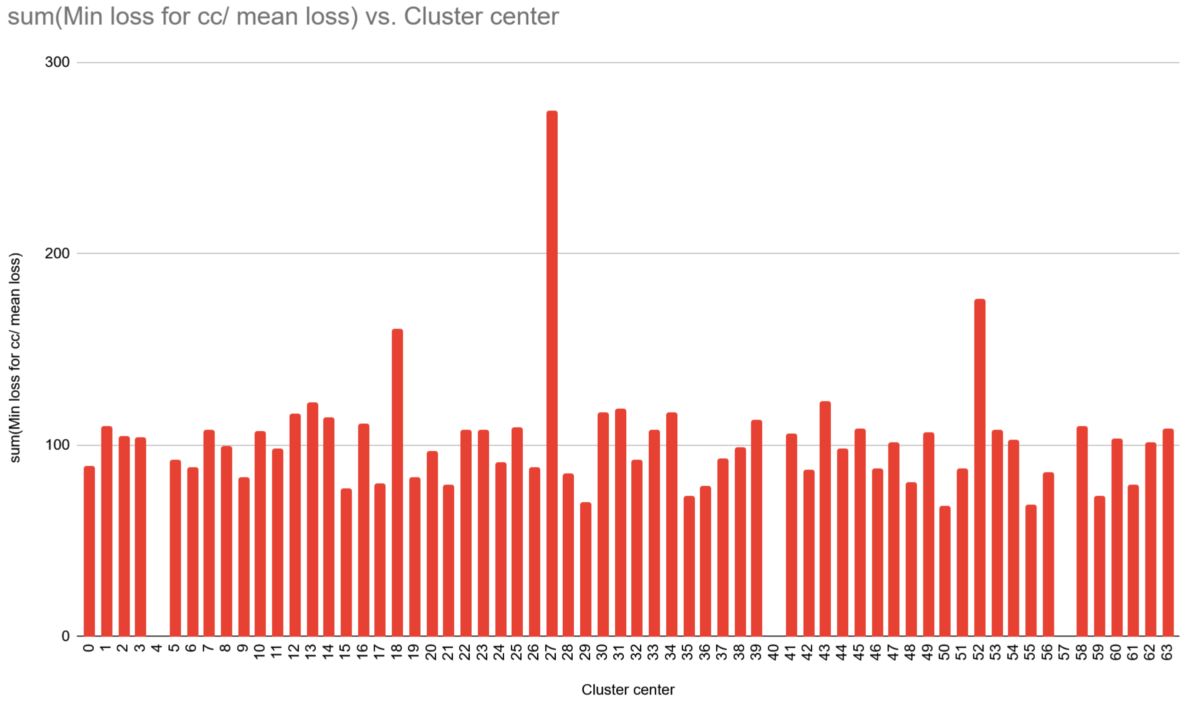

Figure 10. Total log loss for each cell's assigned cluster, normalized it by the mean total log loss. A no-doublet balanced dataset with 64 donors was used.

The outliers with a high number of cell assignments (greater than the median plus 1.5 times the interquartile range) can be considered the culprits that claimed ownership of the low-assignment outliers (less than the median minus 1.5 times the interquartile range). By reinitializing these cluster centers, we should be able to achieve improved clustering results.

In scenarios where the distribution of cells per donor is uneven, it might not be possible to detect outliers using this method. However, empty or near-empty clusters can still be identified by defining an appropriate threshold for cell assignment counts. The corresponding incorrectly merged clusters typically have cc values on the high end. In such cases, we select a number of high-loss clusters equal to the number of empty clusters and treat them as outliers for reinitialization. Even if a correctly clustered center is mistakenly selected as an outlier, the subsequent clustering should not significantly affect the final outcome, as it should be reassigned the same cells.

5.2 Multiple runs with reinitialization and locking.

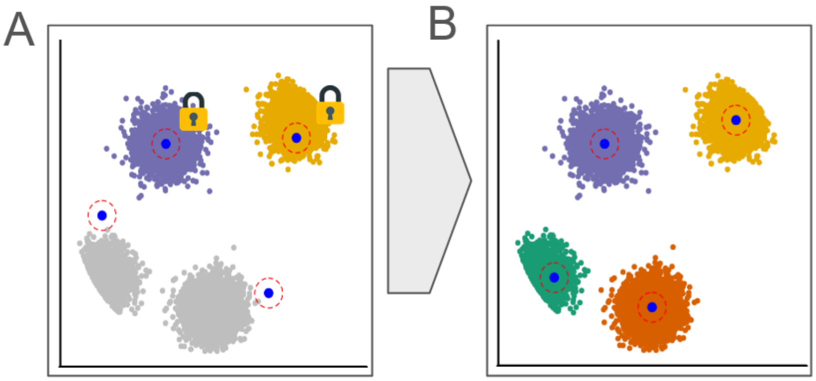

Figure 11. A. After the first clustering run, the bad clusters are detected and reinitialized while good clusters are locked **B.** While the good clusters are locked to updates the KHM clustering is converged.

Now that we can identify poorly formed clusters after algorithm convergence, we reinitialize the corresponding cluster centers and rerun the algorithm to improve clustering accuracy. Since most donors are correctly clustered in the initial run, it is unnecessary to update all the cluster centers. Instead, we lock the cluster centers deemed high quality, effectively freezing them, while allowing only the reinitialized (bad) clusters to update. This strategy is analogous to node freezing and node dropout in machine learning, where parts of a pretrained model remain fixed while selected components are fine-tuned on new data. (Srivastava et al. 2014, Ma et al. 2021). This process can be iteratively repeated, detecting bad clusters and reapplying the locked KHM algorithm with reinitialized centers, until no bad clusters are identified.

6 Results

In this section, we present the results of different methods: vireo, vireo overclustered vireo, souporcell, and souporcell3. Vireo is run using its default configuration. For overclustered vireo, we specify the pre-clustered donors to 10 × k to match the cluster number in our 10x merge setting. Souporcell is run with its default settings. Souporcell3 uses the 10x merge cluster initialization method, the KHM clustering method, and is rerun with reinitialized bad and locked cluster centers. The datasets used are the ones described in supplementary 1.

6.1 Balanced dataset results

6.1.1 Incorrectly merged cluster centers

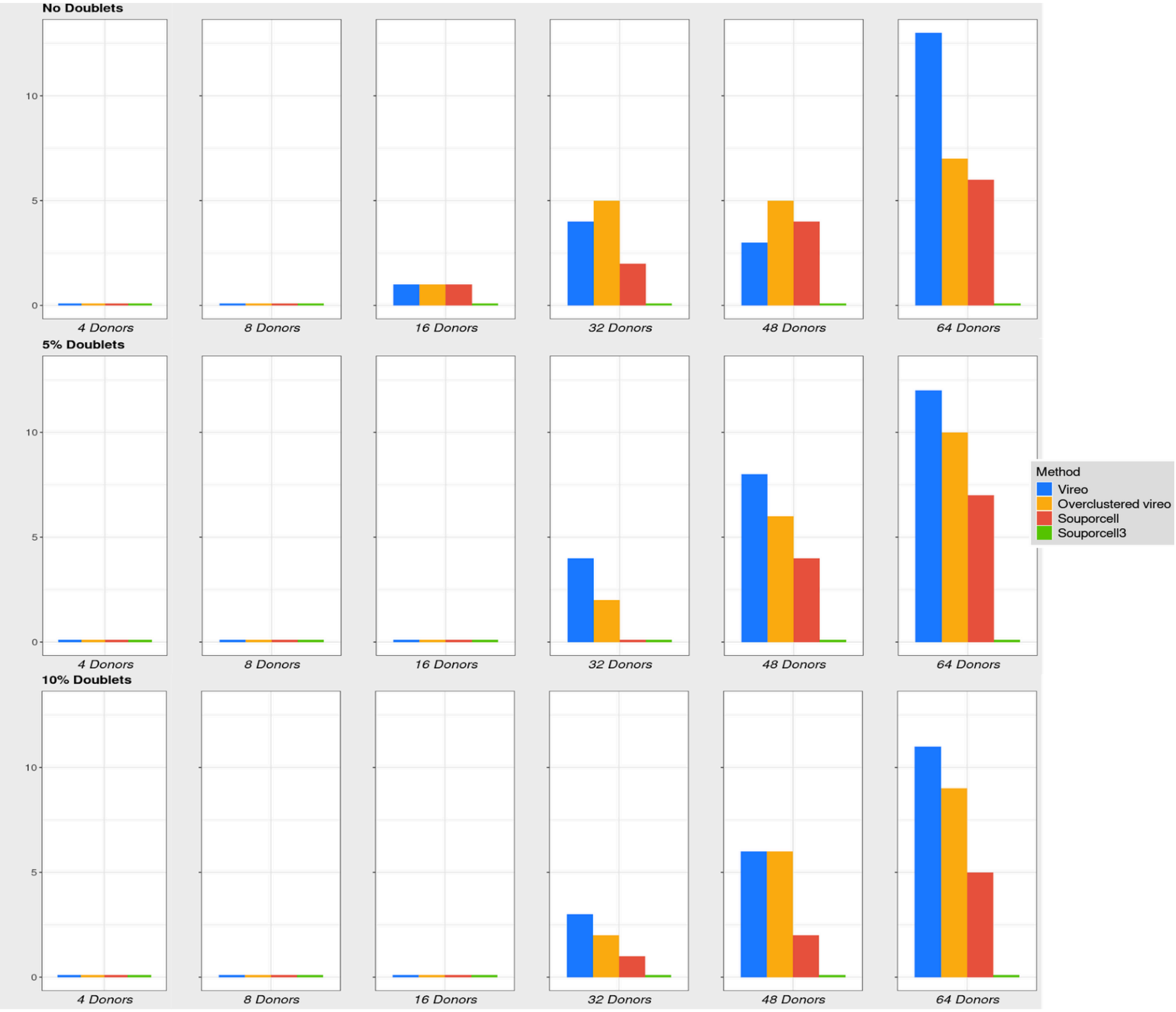

Figure 12. Number of incorrectly merged donor assignments for vireo, overclustered vireo, souporcell, and souporcell3 across different donor sizes and doublet percentages for balanced datasets.

The Figure compares the performance of four demultiplexing tools, vireo, overclustered vireo, souporcell, and souporcell3, based on the number of incorrectly merged clusters they generate when run on datasets with varying numbers of donors (4, 8, 16, 32, 48, 64) under three conditions: no doublets, 5% doublets, and 10% doublets. Incorrectly merged clusters indicate incorrect clustering, where cells from multiple donors are incorrectly clustered into one cluster center; thus, lower values are preferable. Under the no-doublets condition (top panel), all methods show an increasing number of incorrectly merged clusters as the number of donors increases. vireo performs the worst, peaking at 13 incorrectly merged clusters at 64 donors, followed by overclustered vireo and souporcell, while souporcell3 consistently yields no incorrectly merged clusters. With 5% doublets (middle panel), vireo performs slightly better than in the no-doublets scenario, showing 12 incorrectly merged clusters at 64 donors, while souporcell maintains moderate performance with only 7 incorrectly merged clusters. In the most challenging 10% doublet scenario (bottom panel), the pattern persists, vireo and vireo overclustered vireo exhibit a high number of incorrectly merged clusters, souporcell performs moderately, and souporcell3 remains the most robust, maintaining zero incorrectly merged clusters across all donor counts. These results demonstrate that souporcell3 is the most accurate and resilient demultiplexing method across increasing dataset complexity, outperforming both vireo and earlier versions of souporcell.

6.1.2 Adjusted Rand Index

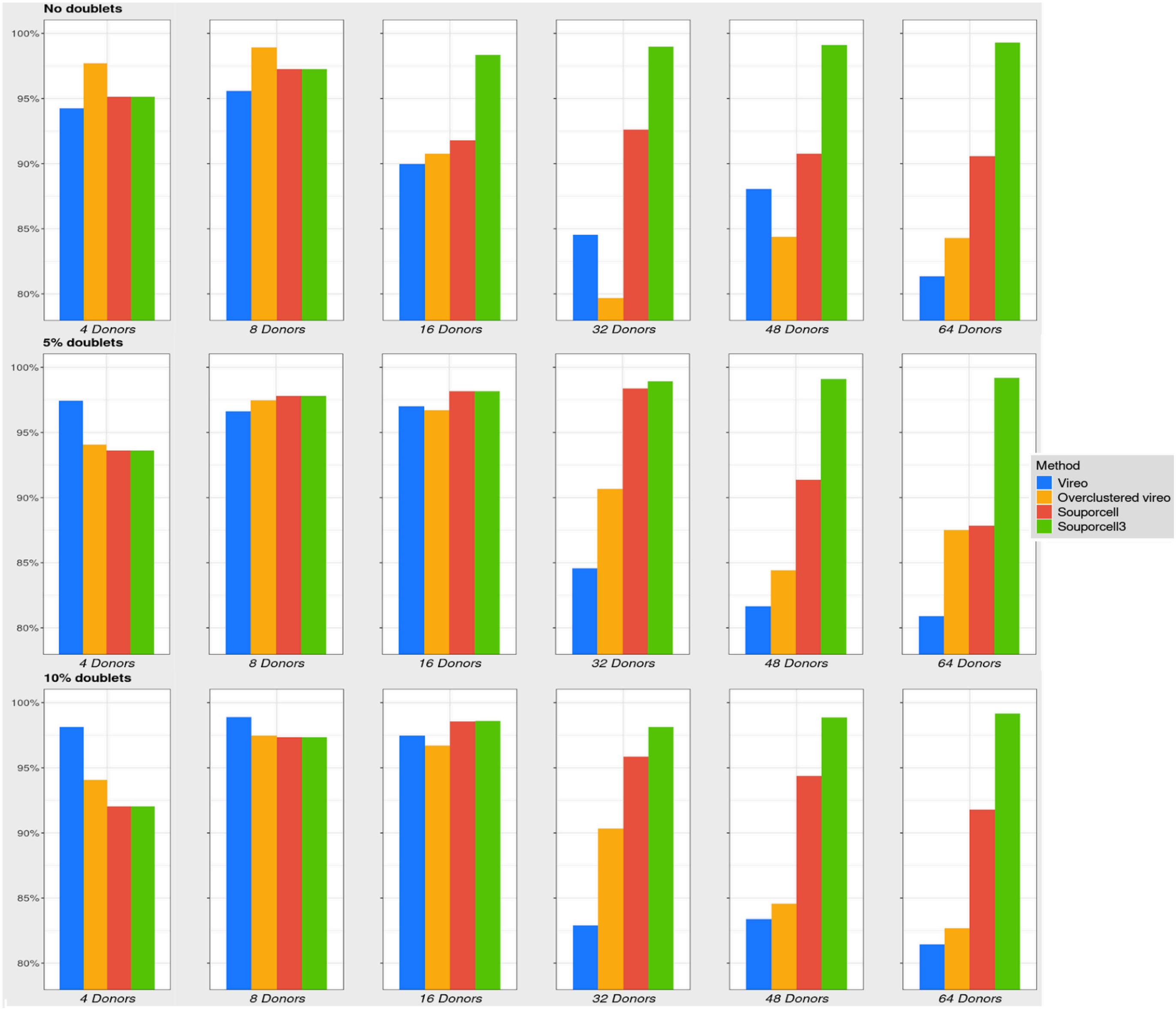

Figure 13. Adjusted Rand Index for vireo, overclustered vireo, souporcell, and souporcell3 across different donor sizes and doublet percentages for balanced datasets.

The figure compares the Adjusted Rand Index, a measure of clustering accuracy, across four demultiplexing methods, vireo, overclustered vireo, souporcell, and souporcell3, on datasets with varying numbers of donors (4, 8, 16, 32, 48, 64) under three conditions: no doublets, 5% doublets, and 10% doublets. In the no-doublet condition (left panel), all methods perform well at low donor counts (4–16 donors), with Rand Index values near or above 0.95. As donor count increases to 32, 48, and 64, vireo's accuracy declines sharply, dropping

below 0.86 at 48 donors and to 0.81 at 64 donors. Overclustered vireo also deteriorates significantly beyond 32 donors, while Souporcell maintains better performance, and Souporcell3 consistently achieves near-perfect clustering (Rand Index > 0.99) across all donor levels. In the 5% doublet condition (middle panel), similar trends emerge: Souporcell3 remains highly accurate across all donor counts, while vireo's performance declines at 64 donors (0.82), and overclustered vireo exhibits a similar drop. The 10% doublet condition (right panel) presents the greatest challenge. Souporcell3 continues to maintain a nearly perfect clustering score, while vireo experiences a pronounced decline in accuracy, especially at 64 donors (Rand Index < 0.79). Souporcell performs similarly to its results in the 5% doublet condition.

The overall trend shows that for datasets with more than 32 donors, overclustered vireo outperforms vireo due to better cluster center selection strategy. However, both Souporcell and Souporcell3 still perform better in comparison. Among these, Souporcell3 consistently outshines the other tools, demonstrating the highest clustering accuracy across all dataset complexities. It maintains near-perfect Adjusted Rand Index values regardless of donor count or doublet rate, making it the most robust and reliable demultiplexing method in this comparison.

6.1.3 Cluster maps for 10% doublet 64 donor balanced dataset

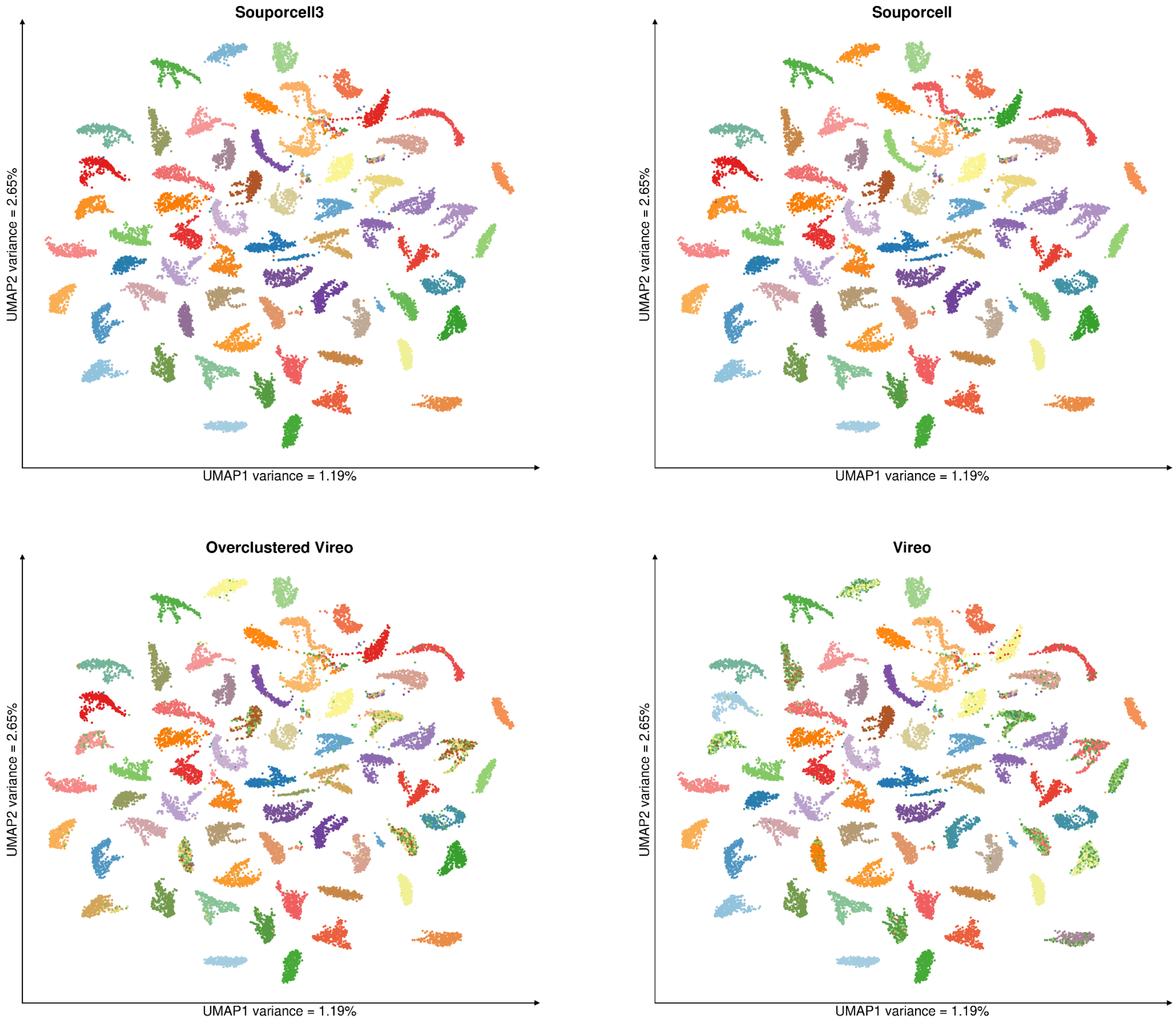

Figure 14. Cluster maps using UMAPs for the x and y axes, showing the result of 10% doublet, 64-donor balanced dataset after running souporcell, souporcell3, vireo, and overclustered vireo prior to doublet detection.

This UMAP plot displays the clustering of single-cell RNA-seq data from a dataset with 64 donors and 10% doublets, with doublets excluded from the visualization as it was generated before doublet detection for all methods. Each point represents a cell and is colored by its ground truth cluster (donor). The UMAP1 and UMAP2 axes capture the primary structure of the high-dimensional cluster data in two dimensions. In the souporcell3 plot, the clusters are well-separated throughout the UMAP space, indicating effective donor demultiplexing. Even in the central region, where many clusters appear close together, they remain distinct and clearly partitioned except for a few outliers, showing that the clustering algorithm is capable of resolving densely packed but separate donor

profiles. In contrast, vireo and overclustered vireo have many ground truth clusters where two or more clusters are assigned.

6.1.4 Heat maps for 10% doublet 64 donor balanced dataset

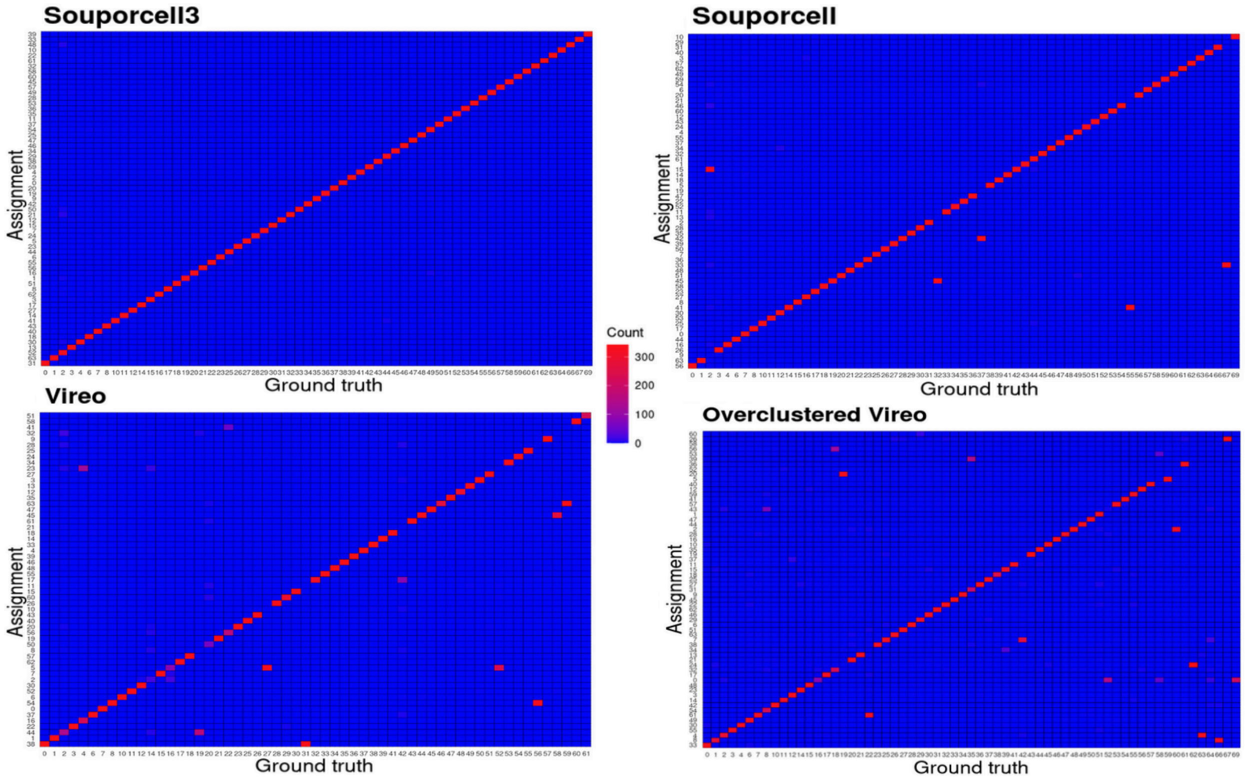

Figure 15. Heat maps showing cell counts for assigned cluster vs ground truth for 64 clusters of 10% doublet balanced dataset after running with souporcell, souporcell3, vireo, and overclustered vireo, prior to doublet detection.

The heat map in figure 15 displays cell counts across 64 clusters for a 64-donor dataset with 10% doublets, result generated using Souporcell3 prior to doublet detection. Each row corresponds to the cluster assignment by the method and each column represents the ground truth clusters, and the color intensity reflects the number of cells assigned, with median cell count per donor being 315. The souporcell3 heatmap shows a strong diagonal pattern, indicating that the majority of cells are correctly assigned to their respective clusters with minimal cross-contamination. This diagonal dominance demonstrates high accuracy in donor assignment, as each cluster predominantly contains cells from a single donor. The other methods all have off diagonal cell counts, meaning there are ground truth clusters where two or more clusters are assigned. The absence of significant off-diagonal cell counts

further supports the effectiveness of Souporcell3 in distinguishing between donors, even in the presence of 10% doublets.

6.2 Unbalanced dataset results

6.2.1 Incorrectly merged cluster centers

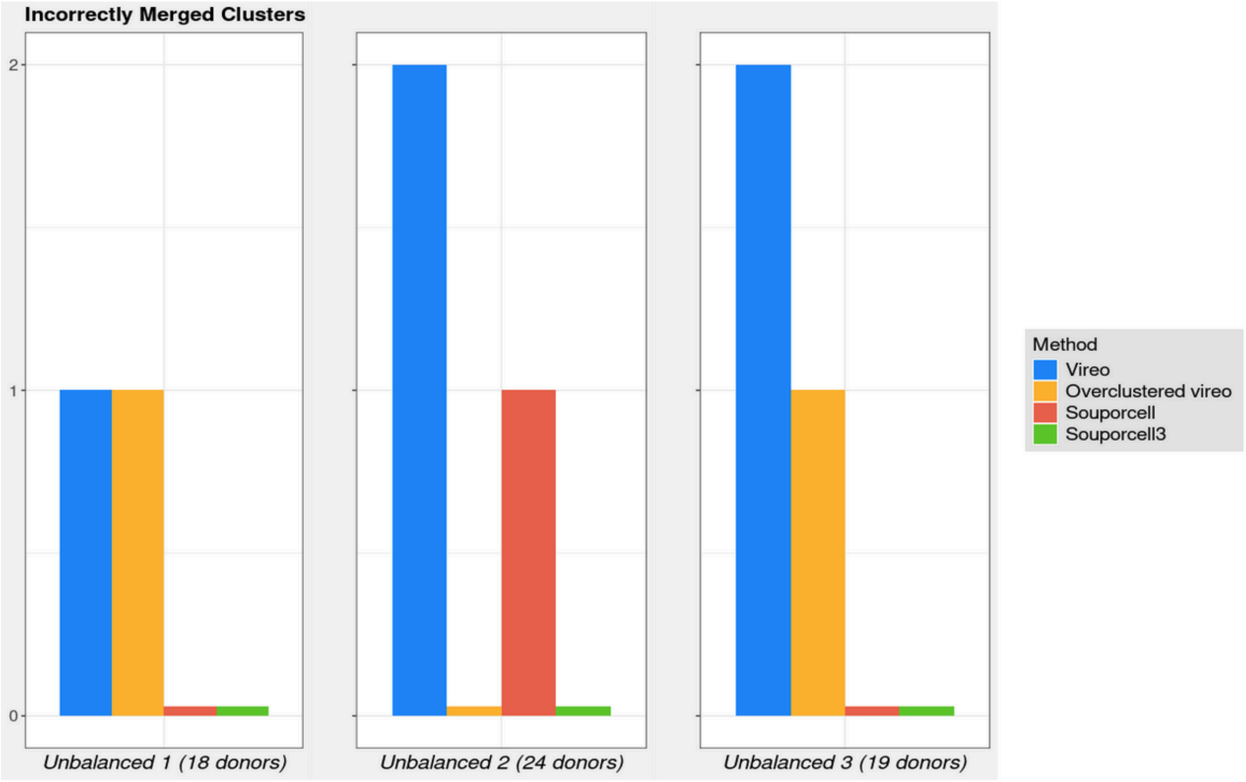

Figure 16. Number of incorrectly merged donor assignments for vireo, overclustered vireo, souporcell, and souporcell3 for unbalanced datasets.

The figure compares four demultiplexing tools, vireo, overclustered vireo, souporcell, and souporcell3, on three unbalanced datasets, showing the number of incorrectly merged clusters. Lower values indicate better performance. Souporcell3 performs perfectly, producing zero incorrectly merged clusters across all datasets. In unbalanced dataset 1, vireo and overclustered vireo each produce a single incorrectly merged cluster. In unbalanced dataset 2, vireo and souporcell each produce incorrectly merged clusters. In the unbalanced dataset 3, vireo produces two incorrectly merged clusters and overclustered vireo produces 1 incorrectly merged cluster, while the other methods remain error-free. Overall, these results show that souporcell3 is the most accurate and robust method across all unbalanced datasets.

6.2.2 Adjusted Rand Index

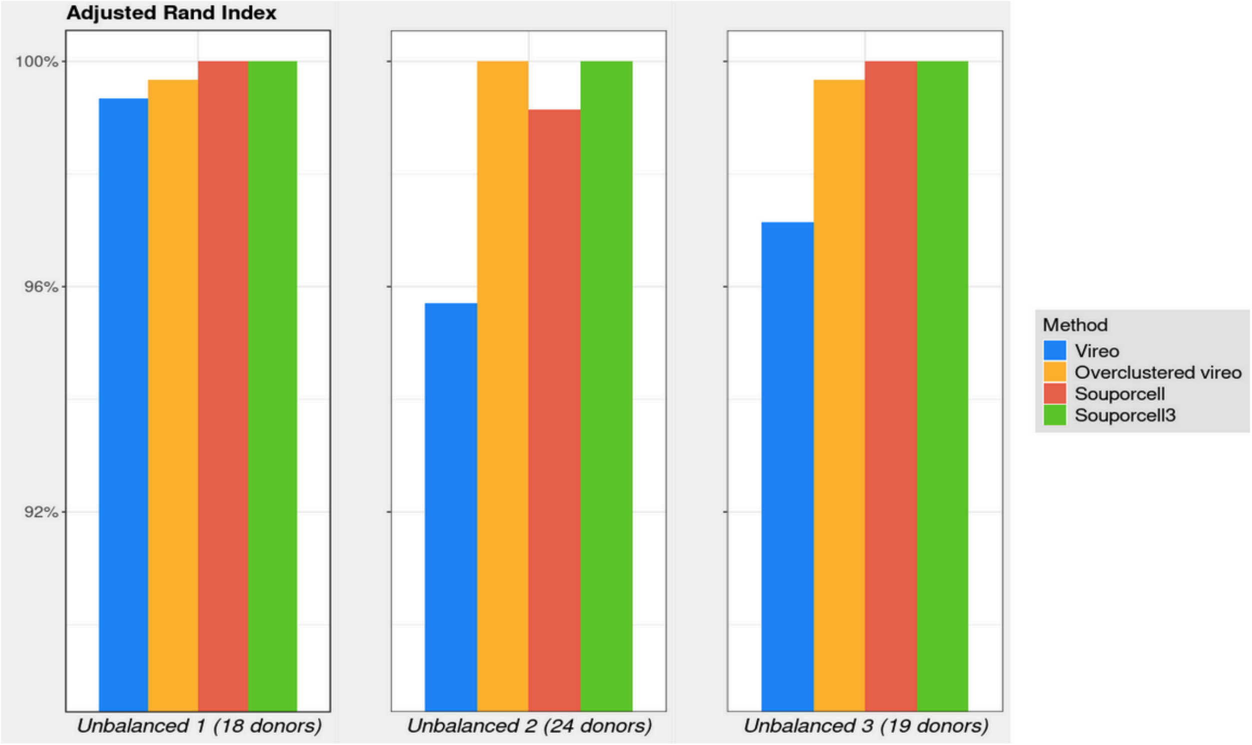

Figure 17. Adjusted Rand Index for vireo, overclustered vireo, souporcell, and souporcell3 for unbalanced datasets.

The figure shows the performance of four demultiplexing tools, vireo, overclustered vireo, souporcell, and souporcell3, on three unbalanced datasets, measured by the Adjusted Rand Index (ARI), where higher values indicate better agreement with the true donor labels. Souporcell3 achieves the highest ARI scores across all datasets, indicating nearly perfect clustering. Overclustered vireo performs slightly worse, with moderately lower ARI values, while vireo shows the lowest ARI, reflecting more frequent clustering errors. These results highlight that souporcell3 provide the most accurate and reliable demultiplexing across unbalanced datasets.

6.2.3 Heat maps

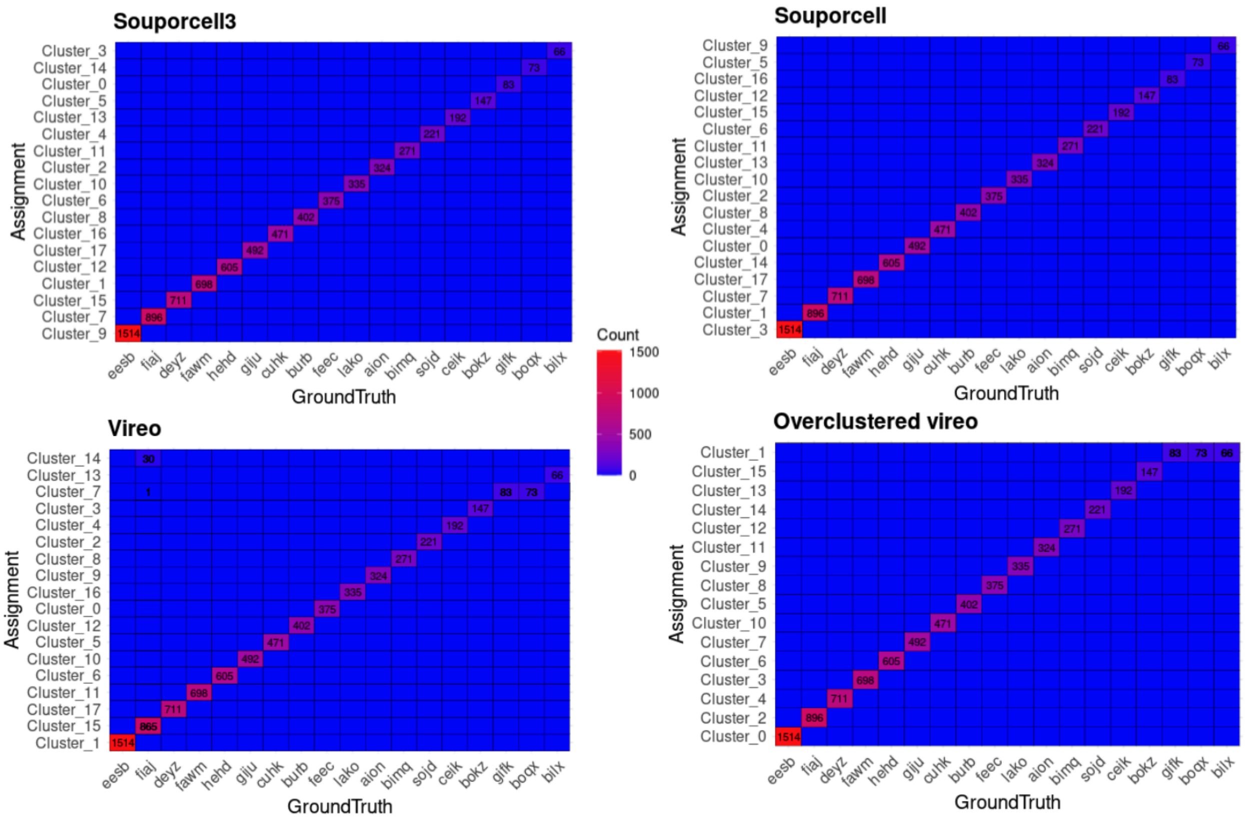

Figure 18. Heat maps showing cell counts for assigned cluster vs ground truth for unbalanced 1 dataset after running with souporcell, souporcell3, vireo, and overclustered vireo.

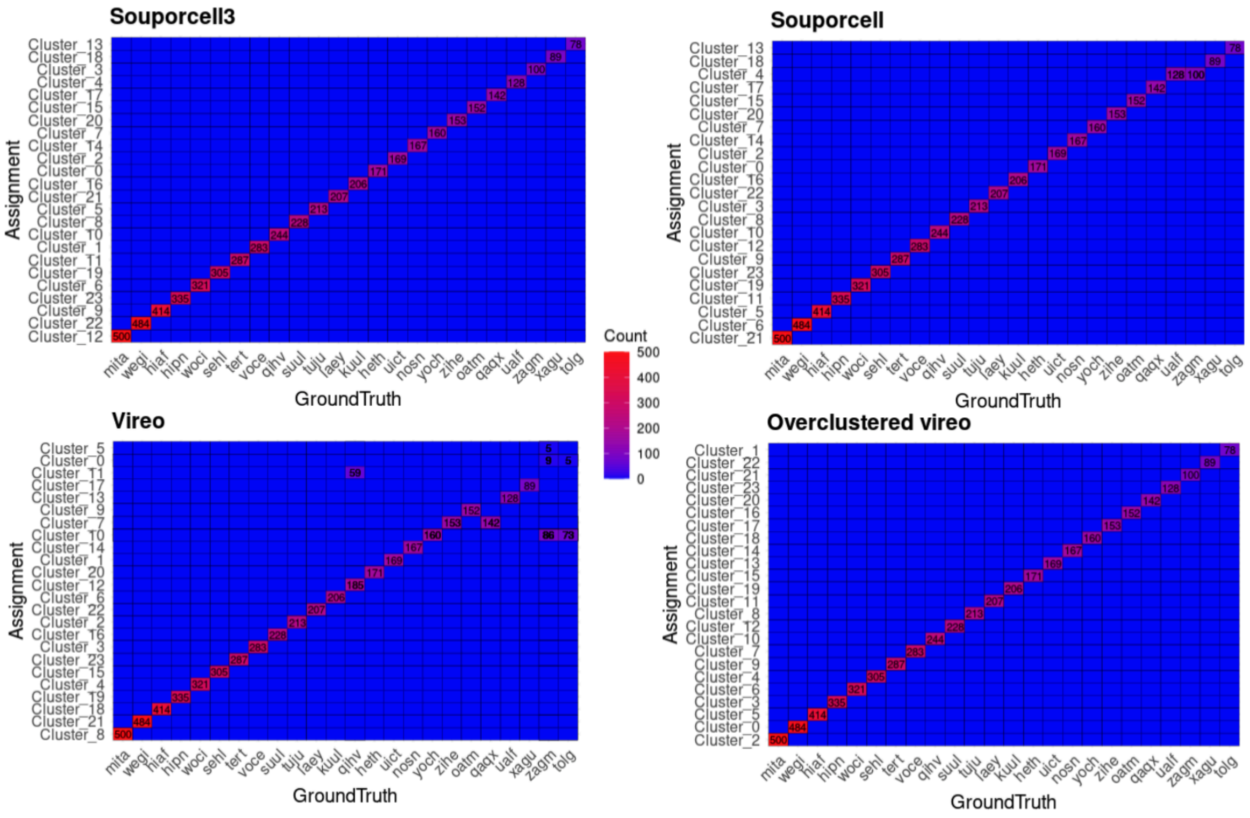

Figure 19. Heat maps showing cell counts for assigned cluster vs ground truth for unbalanced 2 dataset after running with souporcell, souporcell3, vireo, and overclustered vireo.

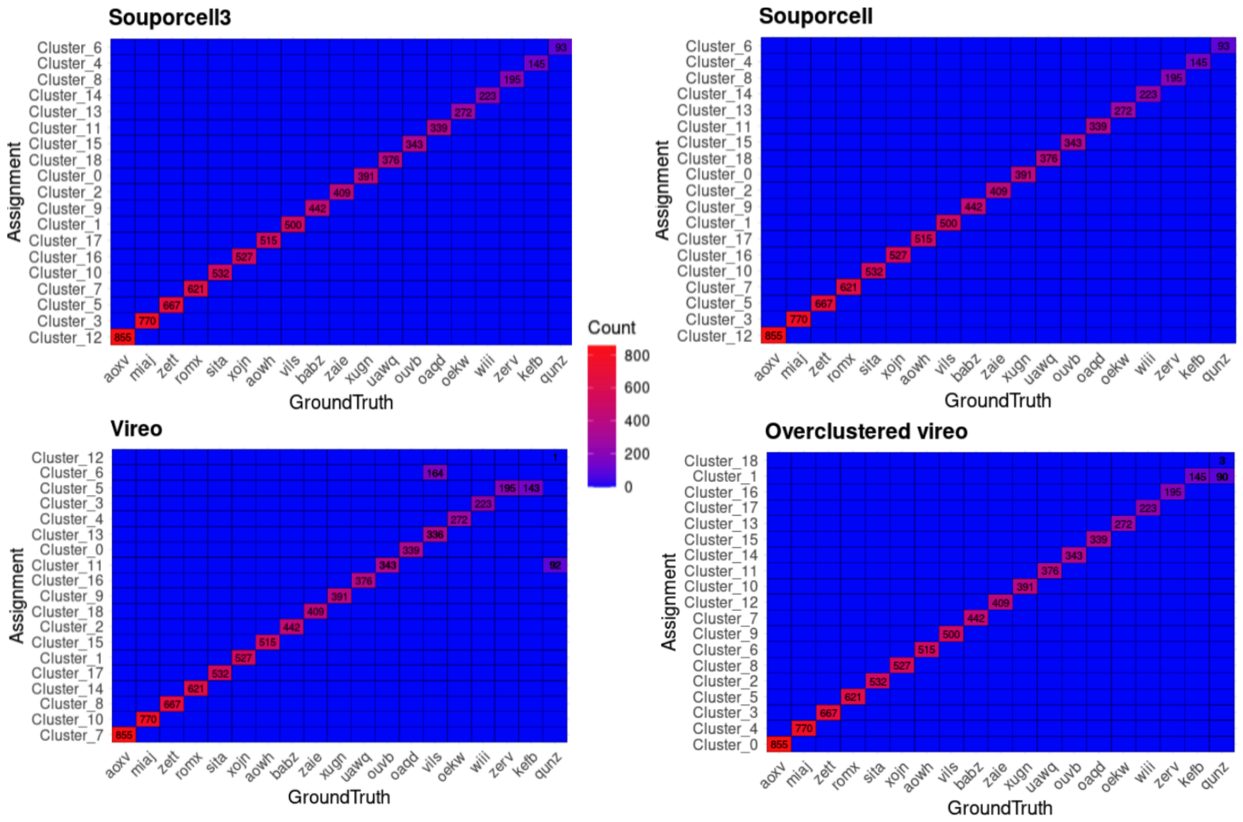

Figure 20. Heat maps showing cell counts for assigned cluster vs ground truth for unbalanced 3 dataset after running with souporcell, souporcell3, vireo, and overclustered vireo.

The figure presents four heatmaps for each of the three unbalanced datasets, comparing the performance of vireo, overclustered vireo, souporcell, and souporcell3. Across all datasets, souporcell3 shows the most accurate clustering, with heatmaps closely matching the true donor labels and minimal mixing between clusters. Souporcell also performs well, with only minor deviations in a few clusters. In contrast, vireo and overclustered vireo show more mixing and less clearly defined clusters in the heatmaps. These results demonstrate that souporcell3 provides the most precise and robust demultiplexing across all unbalanced datasets.

References

10X Genomics (2020) *What is the maximum number of cells that can be profiled?* [online] Available at: https://kb.10xgenomics.com/hc/en-us/articles/360001378811-What-is-the-maximum-number-of-cells-that-can-be-profiled [Accessed 3 Jun. 2025].

Aloise, D., Deshpande, A., Hansen, P. & Popat, P. (2009). NP-hardness of Euclidean sum-of-squares clustering. *Machine Learning*, 75(2), 245–248. https://doi.org/10.1007/s10994-009-5103-0

Arthur, D. & Vassilvitskii, S. (2007). *k-means++: The advantages of careful seeding*. In: *Proceedings of the Eighteenth Annual ACM-SIAM Symposium on Discrete Algorithms (SODA)*. Society for Industrial and Applied Mathematics, New Orleans, Louisiana, pp. 1027–1035.

Cillo, A.R., Kürten, C.H.L., Tabib, T., Qi, Z., Onkar, S., Wang, T., et al. (2020) *Immune landscape of viral- and carcinogen-driven head and neck cancer*. Immunity, 52(1), 183–199.e6.

Chung HM, Huang Y. SNPmanifold: detecting single-cell clonality and lineages from single-nucleotide variants using binomial variational autoencoder. *Genome Biol* 2025;**26**:309.

European Nucleotide Archive (ENA) (2020a) *Sample accessions ERS2630502–ERS2630507 for individual cell lines euts, nufh, babz, oaqd, and ieki*. Available at: https://www.ebi.ac.uk/ena/browser/view/ERS2630502~ERS2630507

European Nucleotide Archive (ENA) (2020b) *Sample accessions SAMEA6833385*. Available at: https://www.ebi.ac.uk/ena/browser/view/SAMEA6833385

European Nucleotide Archive (ENA) (2020c) *Sample accessions SAMEA6833405*. Available at: https://www.ebi.ac.uk/ena/browser/view/SAMEA6833405

European Nucleotide Archive (ENA) (2020d) *Sample accessions SAMEA6833432*. Available at: https://www.ebi.ac.uk/ena/browser/view/SAMEA6833432

Güngör, Z. & Ünler, A. K-harmonic means data clustering with simulated annealing heuristic. Appl. Math. Comput. 184, 199–209 (2007).

Güngör, Z. & Ünler, A. K-harmonic means data clustering with tabu-search method. Appl. Math. Model. 32, 1115–1125 (2008).

Huang, Y., McCarthy, D.J. and Stegle, O. (2019). Vireo: Bayesian demultiplexing of pooled single-cell RNA-seq data without genotype reference. Genome Biology, 20(1), p.273. https://doi.org/10.1186/s13059-019-1865-2

Hubert, L., Arabie, P. Comparing partitions. *Journal of Classification* **2**, 193–218 (1985). https://doi.org/10.1007/BF01908075

Human Cell Atlas (2020a) *Immune Landscape of Viral- and Carcinogen-Driven Head and Neck Cancer Project*. Available at: https://explore.data.humancellatlas.org/projects/3089d311-f9ed-44dd-bb10-397059bad4dc.

Human Cell Atlas (2020b) *Human Cell Atlas Project: cc95ff89-2e68-4a08-a234-480eca21ce79*. Available at: https://explore.data.humancellatlas.org/projects/cc95ff89-2e68-4a08-a234-480eca21ce79.

Human Cell Atlas (2021) *Mapping the Developing Human Immune System Across Organs Project*. Available at: https://explore.data.humancellatlas.org/projects/fcaa53cd-ba57-4bfe-af9c-eaa958f95c1a.

Inaba, M., Katoh, N. & Imai, H. (1994). Applications of weighted Voronoi diagrams and randomization to variance-based k-clustering. In: *Proceedings of the Tenth Annual Symposium on Computational Geometry (SCG '94)*. ACM Press, New York, pp. 332–339. https://doi.org/10.1145/177424.178047

Jardine, L., Wiscombe, S., Reynolds, G., McDonald, D., Fuller, A., Green, K., et al. (2021) *Mapping the developing human immune system across organs*. Science, 373(6558), eabh1516.

Jerber J, Seaton DD, Cuomo ASE *et al.* Population-scale single-cell RNA-seq profiling across dopaminergic neuron differentiation. *Nat Genet* 2021;**53**:304–12.

Kiselev, V.Y. et al. SC3: consensus clustering of single-cell RNA-seq data. Nat. Methods 14, 483–486 (2017). https://doi.org/10.1038/nmeth.4236

Ma, X., Li, Z., Zhang, X., Wang, H. and Wang, X. (2021). Few-shot Learning for Named Entity Recognition Based on BERT and Two-level Model Fusion. ACM Transactions on Asian and Low-Resource Language Information Processing (TALLIP), 20(3), pp.1–19. https://doi.org/10.1145/3440750

McGinnis, C.S., Murrow, L.M. and Gartner, Z.J. (2019). DoubletFinder: Doublet detection in single-cell RNA sequencing data using artificial nearest neighbors. Cell Systems, 8(4), 329–337.e4. https://doi.org/10.1016/j.cels.2019.03.003.

Peizhuang, W. Pattern Recognition with Fuzzy Objective Function Algorithms (James C. Bezdek). Philadelphia 25, 1 (1983).

Rand, W.M., 'Objective criteria for the evaluation of clustering methods', *Journal of the American Statistical Association*, vol. 66, no. 336, 1971, pp. 846-850.

Srivastava, N., Hinton, G., Krizhevsky, A., Sutskever, I. and Salakhutdinov, R. (2014). Dropout: A Simple Way to Prevent Neural Networks from Overfitting. Journal of Machine Learning Research, 15(1), pp.1929–1958.

Wolock, S.L., Lopez, R. and Klein, A.M. (2019). Scrublet: computational identification of cell doublets in single-cell transcriptomic data. Cell Systems, 8(4), 281–291.e9. https://doi.org/10.1016/j.cels.2018.11.005

Xu J, Falconer C, Nguyen Q *et al.* Genotype-free demultiplexing of pooled single-cell RNA-seq. *Genome Biol* 2019;**20**:290.

Zheng, G.X.Y. et al. (2017). Massively parallel digital transcriptional profiling of single cells. Nature Communications, 8, 14049. https://doi.org/10.1038/ncomms14049

Zhang, B., Hsu, M. & Dayal, U. K-harmonic means—a data clustering algorithm. Hewlett-Packard Labs Technical Report HPL-1999-124, 55 (1999).

Zhang, B. Generalized K-harmonic means – dynamic weighting of data in unsupervised learning. In: Proceedings of the 2001 SIAM International Conference on Data Mining, Society for Industrial and Applied Mathematics, Philadelphia, PA (2001).
